# Temporal changes in the effects of ambient temperatures on hospital admissions in Spain

**DOI:** 10.1371/journal.pone.0218262

**Published:** 2019-06-13

**Authors:** Èrica Martínez-Solanas, Xavier Basagaña

**Affiliations:** 1 ISGlobal, Barcelona, Spain; 2 Universitat Pompeu Fabra (UPF), Barcelona, Spain; 3 CIBER Epidemiología y Salud Pública (CIBERESP), Madrid, Spain; Columbia University, UNITED STATES

## Abstract

**Background:**

The exposure to extreme ambient temperatures has been reported to increase mortality, although less is known about its impact on morbidity. The analysis of temporal changes in temperature-health associations has also focused on mortality with no studies on hospitalizations worldwide. Studies on temporal variations can provide insights on changes in susceptibility or on effectiveness of public health interventions. We aimed to analyse the effects of temperature on cause-specific hospital admissions in Spain and assess temporal changes using two periods, the second one characterized by the introduction of a heat health prevention plan.

**Methods:**

Daily counts of non-scheduled hospital admissions for cardiovascular, cerebrovascular and respiratory diseases and daily maximum temperature were obtained for each Spanish province for the period 1997–2013. The relationship between temperature and hospitalizations was estimated using distributed lag non-linear models. We compared the risk of hospitalization due to temperatures (cold, heat and extreme heat) in two periods (1997–2002 and 2004–2013).

**Results:**

Cold temperatures were associated with increased risk of cardiovascular, cerebrovascular and respiratory hospital admissions. Hot temperatures were only associated with higher hospital admissions for respiratory causes while hospitalizations for cardiovascular and cerebrovascular diseases did not increase with heat. There was a small reduction in heat-related respiratory admissions in period 2. Whereas cold-related hospitalizations for cardiovascular and cerebrovascular diseases increased in period 2, a significant reduction for respiratory hospitalizations was reported.

**Conclusions:**

Our results suggested that heat had an adverse impact on hospital admissions for respiratory diseases, while cold increased the risk of the three studied cause-specific hospitalizations. Public health interventions should also focus on morbidity effects of temperature.

## 1. Introduction

Exposure to extreme ambient temperatures is an important public health hazard. The most reported health impacts of extreme temperatures have been increases in mortality, especially among the most vulnerable individuals such as the elderly and those with prior medical conditions [1,2]. A large study conducted in 13 countries estimated that around 8% of deaths can be attributable to temperatures [3]. In Spain this number was 6.5% and the highest mortality was associated with cold temperatures.

While the effects of temperatures on mortality have been well documented, less is known about their impact on morbidity. Different outcomes have been examined, such as hospital admissions [4–8], emergency department visits [9–13], outpatient visits [14] or general practice (GP) visits [15], with inconsistent results. For example, an overview of systematic reviews including twenty articles reported no significant heat effects for cardiovascular and cerebrovascular morbidity after conducting a meta-analyses, while a positive association with heat was found for respiratory morbidity among the elderly [16]. This was in line with another systematic review, where authors found an increase of 3.2% in respiratory hospitalizations with a 1°C increase during hot days, but uncertain results for cardiovascular hospitalizations [17]. For the case of cold temperatures, results of a small number of studies reported no clear effects for cardiovascular diseases. An increased risk in respiratory diseases with cold was observed in different systematic reviews [18,19]. Overall, clear associations of ambient temperature have been shown for respiratory diseases, while for cardiovascular and cerebrovascular hospitalizations there was no clear evidence of their relationship with ambient temperatures. Therefore, given the inconsistency of the studies available, more research is needed to better understand the impact of low and high ambient temperatures on morbidity.

Previous research focused on the effects of temperature in a specific city or a region [7,20,21], but less common is the analysis of a whole country. Only one study examined the effects of temperature using a national register of all hospital admissions in England and Wales, but this study was restricted to patients admitted for myocardial infarction (MI) and other acute coronary syndromes (ACS) [22]. In terms of studies conducted in Spain, Michelozzi et al. evaluated the impact of high temperatures on hospital admissions in twelve European cities, including one in Spain, Barcelona [4]. The authors found a positive association for respiratory causes (mainly in the oldest age group), while in most cities they did not detect any association for cardiovascular and cerebrovascular admissions. A study conducted in Madrid found a slight increase in hospital admissions for respiratory diseases at high temperatures and no association was found for cardiovascular causes [23]. In the Catalonia region, Ponjoan et al. reported higher incidence of cardiovascular hospitalizations for cold spells, though no effect was seen for heat waves [24].

Several factors can modify the vulnerability to extreme temperatures, such as a pure biological adaptation [25], improvements in healthcare system and housing conditions [26], and the implementation of public health adaptation measures that reduce the vulnerability to cold and heat [27]. One way to study the potential adaptation to extreme temperatures is looking at temporal variations in temperature-health associations. This kind of studies also allows testing the health impact of changes in temperature distribution. Some studies have addressed this issue in terms of temperature-related mortality [28–30]. The authors found a decline in mortality due to heat in the last decades in some countries, like Spain, but not in all regions included. For cold, a multi-country study observed different trends in mortality depending on the country [30]. In the case of Spain, the authors reported a decrease in cold-related mortality. Despite the importance of temporal variation in temperature-health associations, to the best of our knowledge there are no studies looking at such trends in terms of morbidity.

The comparison of the effects of temperature on health in different periods can be a useful tool for assess the effectiveness of public interventions. In order to prevent the health effects of heat, the majority of European countries implemented Heat Health Prevention Plans (HHPP), after the devastating effects of the 2003 European heat wave [31]. That was the case of Spain, where the “National plan for preventive actions against the effects of excess temperatures on health" [32] was put in place in 2004. The Health Ministry is the responsible of the plan, but it is characterized by the coordination between different institutions, such as some National ministries (Home Office; Agriculture, Food and Environment; Justice), regional departments of Health and Social Services, Spanish social organizations as well as municipalities. The main objective of the plan is to coordinate the different institutions involved in the execution of the plan and also to establish actions and strategies to reduce the health effects of heat waves. The National Meteorology Agency establishes thresholds to activate different actions based on weather forecasts. Such actions include dissemination of preventive information to the general population and specific high-risk groups, such as the elderly, and the activation of a general helpline. It is also assured that there are enough medical and health professionals to attend possible emergencies derived from high temperatures during episodes of heat wave. The plan also gives information to health professionals and emergency and social services. For instance, in some areas of Spain, GPs are responsible for creating a census of vulnerable people to be followed during heat wave episodes.

The evaluation of the effectiveness of these public health interventions is important to prevent the short-term negative health effects of temperatures and improve adaptation to future climate warming. However, such evaluations are complex [27,33,34]. The majority of existing studies compare the effects of heat before and after the implementation of these plans, focusing mainly on changes in temperature-related mortality. In general, studies observed a reduction of the effect of high temperatures on mortality after the implementation of prevention programs [35–37]. However, there are only a very few studies assessing the impact of these public interventions in reducing morbidity effects. The analysis of the effect of the preventive plans on morbidity can provide new insights that can lead to establishing more actions, such us strengthening hospital services, if needed.

Therefore, the objectives of this study were 1) to analyse the impact of ambient temperatures on hospital admissions for cardiovascular, cerebrovascular and respiratory diseases over a 17-year period in Spain; and 2) to assess the temporal changes in the effects of cold, heat and heat waves using two periods (1997–2002 and 2004–2013), the second one characterized by the introductions of a HHPP.

## 2. Methods

### 2.1. Setting

This study was carried out in Spain, a country with a population of 46.5 million living in 50 provinces (excluding Ceuta and Melilla in North Africa). Spain has mainly a Mediterranean climate, with dry, hot summers and winters with balanced temperatures and low rainfall. However, due to its geographical situation, other climates are present, such as oceanic (north-western region), arid and semi-arid (south-western region), subtropical (Canary Islands) and continental climate (mountain ranges). The study period covered 17 years from 1997 to 2013.

### 2.2. Data

#### 2.2.1. Hospital admissions

Daily counts of non-scheduled hospital admissions registered in Spain during the study period were obtained from *Conjunto Mínimo Básico de Datos* (CMBD) of the *Instituto de Información Sanitaria* (Spanish Ministry of Health, Social Services and Equity). This register, with data available since 1997, is compulsory for all hospitals from the National Health System, even though private hospitals have been included during the last years. Currently, 92% of the hospitalizations in Spain are gathered in this register and 15.9% correspond to private sector [38]. This register provides individual data with information of date and province of hospitalization (regardless of their residence), sex, age (which we grouped as all ages, 16–64, 65–74, 75–85, 85 or more) and cause of hospitalization. These age groups were selected considering that the highest vulnerabilities have been reported among the elderly [1]. We aggregated hospitalization counts by province and we selected three specific causes based on previous research [4,6,16–19,39]: cardiovascular diseases (ICD-10-CM codes: I00 –I99), cerebrovascular diseases (ICD-10-CM codes: I60 –I69) and respiratory diseases (ICD-10-CM codes: J00 –J99).

#### 2.2.2. Weather

Daily maximum and minimum temperature registered during 24 hours was obtained for each provincial capital from the European Climate Assessment & Dataset (ECA&D project) [40]. We assigned temperature of the province capital station to all municipalities in the province. We imputed missing values in temperature variables (0.01% of the data) according the following criteria: 1) the mean temperature registered on the day after and before the missing day, if only one value was omitted; or 2) the temperature registered in the most correlated station for the same day-month-year, if more than two consecutive days were missing. Our main results did not change if missing values were excluded (data not shown).

In order to assess differences in temperature-related hospitalizations in period 2, after the implementation of the HHPP, we made use of the official criteria for the activation of the Plan on a given day. These thresholds were established based on both maximum and minimum temperatures registered in each capital of the province. In general, they correspond to the 95th percentile of the historical series of summer maximum and minimum temperatures [32]. The Spanish HHPP defines four risk levels to classify hot days depending on the number of forecasted days exceeding the thresholds in the next 5 days (level 0: no exceedances; level 1: 1–2 days; level 2: 3–4 days; level 3: 5 days). We defined plan activation as days that would fall into levels 1 to 3 of the HHPP.

### 2.3. Statistical analysis

We used distributed lag non-linear models (DLNM) to estimate the relationship between temperature and hospital admissions. This methodology, described elsewhere [41], allows capturing potentially non-linear exposure-response functions, and capturing delayed effect. We modelled the association using two stages. Firstly, analyses were conducted in each province separately. Then, province-specific estimations were combined using multivariate meta-analysis.

#### 2.3.1. First stage: Time series models for province

In order to estimate the location-specific temperature-hospitalizations association, we fitted a time-series quasi Poisson regression model separately in each province. The exposure-response association was modelled using a quadratic B-spline with 3 internal knots placed at the 10th, 50th and 90th percentiles of location-specific temperature distribution [42]. We controlled for seasonality by including a natural cubic B-spline of day of the year with equal spaced knots and 8 degrees of freedom per year. An interaction between this spline function and indicator of year was specified to relax the assumption of a constant seasonal trend. Long-term trends were controlled by including a linear term for year. In addition, we included an indicator of day of the week, holidays and the number of hospitalizations due to influenza (except when conducting analyses for respiratory diseases). The lag-response association was modelled by a natural cubic B-spline with an intercept and three internal knots placed at equally spaced values in the log scale. The Poisson regression model was given as follows:

$$Log\;E\;\left(Y_{t}\right)=intercept+cb+dow+hday+hosp\_influenza+S1\left(dos,\;df=8\right):factor\left(year\right)+S2\left(time,\;df=1\;per\;decade\right)$$

where Y_t_ corresponds to the series of daily mortality counts, cb the cross-basis matrix of temperature produced by DLNM, dow to an indicator variable for the day of the week, hday to an indicator for holidays, hosp_influenza to the number of hospitalizations due to influenza, S1 to a quadratic B-spline of the day of the season, factor to an interaction term by year and S2 to a natural cubic B-spline of year.

To include possible long delays in the effects of temperature, the lag period was extended up to 21 days to capture long lags related to cold [3]. The results of this stage, cumulated over all lags to obtain the overall temperature-hospitalizations association curve, were reported as percent changes.

We performed sensitivity analyses to evaluate the association of temperatures and hospitalizations considering the exclusion of year 2003, changes in the lag period, and modification of seasonality modelling (S5 and S9 Tables).

#### 2.3.2. Second stage: Meta-analysis

In the second stage of the analysis, we pooled the estimated location-specific associations using a multivariate meta-regression model. We derived the best linear unbiased prediction of the overall cumulative exposure-response association in each location. This approach allows areas with small daily hospitalizations counts or short series, usually characterized by very imprecise estimates, to borrow information from larger populations [3]. The pooled curve was used to define the temperature percentile of minimum hospitalizations (MHP).

#### 2.3.3. Temporal variations

In order to assess temporal variations in the relationship between temperature and hospitalizations, we split the study period into two, period 1 from 1997–2002 and period 2 from 2004–2013. We excluded year 2003 from the analysis because of the unusually high temperatures registered in that summer (average daily maximum temperature of 36.7°C) (S1 Table). These two periods were defined in order to evaluate, using a before-after comparison, the Spanish HHPP implemented in 2004. To compare the two periods, we used the curve for each period resulting from the second stage of the analysis. A Wald test was used to test differences between temperature-related cause-specific hospitalizations in period 1 and period 2 [28]. We also computed the relative risk at the 1st and 99th percentiles using the MHP as the reference, reported as extreme cold and extreme heat effects, respectively. The results were reported as percent differences. All analyses were conducted for the three causes of hospitalization and results were stratified by sex and age group.

As the Spanish plan paid particular attention to heat wave periods, we calculated the risk associated with heat waves, according to the definition used in the HHPP. Hence, in order to compare the two periods, we restricted the analyses from June 1st to September 15th, the period in which the plan was activated. A daily binary variable was created to indicate if the plan was or would have been activated (for the first period, in which the plan did not exist) or not, according to the temperatures registered during five consecutive days. This variable was included in the first-step model instead of temperature. Seasonality was restricted to 3 degrees of freedom per year. We then performed a univariate meta-analysis of the province-specific coefficients.

Since a previous study found that the thresholds in the Spanish HHPP did not capture all the effects of heat on mortality [43], we also conducted sensitivity analyses using other definitions of heat waves based on relative thresholds of historical series of daily maximum temperature. In particular, we defined heat waves as periods of ≥2, ≥3 or ≥4 consecutive days with maximum temperature exceeding the 90th, 92.5th, 95th or 97.5th percentile of the province, as used in previous papers [44,45]. These results are reported in the Supporting Information (S8 Table).

All analyses were performed with R software (version 3.3.3). The following packages were used: dlnm (version 2.3.2) and mvmeta (version 0.4.7). The code used is available on https://github.com/ericamartinez/Temperatures_Hospitalizations.

## 3. Results

Overall, 37,278,921 hospital admissions were registered in Spain during the study period. Restricting to the three selected causes, the number of hospitalizations was 10,550,849. Cardiovascular and respiratory diseases were the major causes of hospitalization (around 12% and 13%, respectively), while admissions for cerebrovascular diseases accounted for 3.5% of hospitalizations (Table 1). Cardiovascular and cerebrovascular hospitalizations were sex-balanced, while men suffered more hospitalizations due to respiratory diseases (Table 1). In terms of age, the three causes analysed had a similar distribution. Barcelona and Madrid were the provinces that registered the highest percentage of hospitalizations (S2 Table). An important climatic variability was observed among the Spanish provinces, with a daily mean temperature ranging from 16.9° in Leon to 25.7° in Sevilla (S2 Table). The distribution of all-cause hospital admissions and daily maximum temperature by month and day of the week is reported in Supporting Information (S3 Table).

**Table 1:** Descriptive statistics on daily number of hospital admissions in Spain by causes, sex and age group (1997–2013).

	Total number of hospital admissions	% of hospital admissions	Daily hospital admissions		
			Mean	Min.	Max.
**TOTAL**	37,278,921		6,004.0	22	9,090
**CARDIOVASCULAR DISEASES**					
**Sex**					
Women	1,976,979	44.2	318.4	0	589
Men	2,498,724	55.8	402.4	3	681
Total	4,475,984	100.0	720.9	3	1,212
**Age group**					
16–64	1,132,477	25.3	182.4	1	289
65–74	1,106,467	24.7	178.2	0	332
75–84	1,498,936	33.5	241.4	1	472
> = 85	716,250	16.0	115.4	0	295
**CEREBROVASCULAR DISEASES**					
**Sex**					
Women	618,810	46.9	99.7	0	163
Men	701,504	53.1	113.0	0	178
Total	1,320,418	100.0	212.7	0	320
**Age group**					
16–64	296,462	22.5	47.7	0	87
65–74	330,284	25.0	53.2	0	96
75–84	471,232	35.7	75.9	0	131
> = 85	219,521	16.6	35.4	0	83
**RESPIRATORY DISEASES**					
**Sex**					
Women	1,772,426	37.3	285.5	0	902
Men	2,981,517	62.7	480.2	0	1,350
Total	4,754,447	100.0	765.7	2	2,245
**Age group**					
16–64	1,015,935	21.4	163.6	0	513
65–74	881,821	18.5	142.0	0	498
75–84	1,307,356	27.5	210.6	0	733
> = 85	742,499	15.6	119.6	0	540

**Fig 1:**
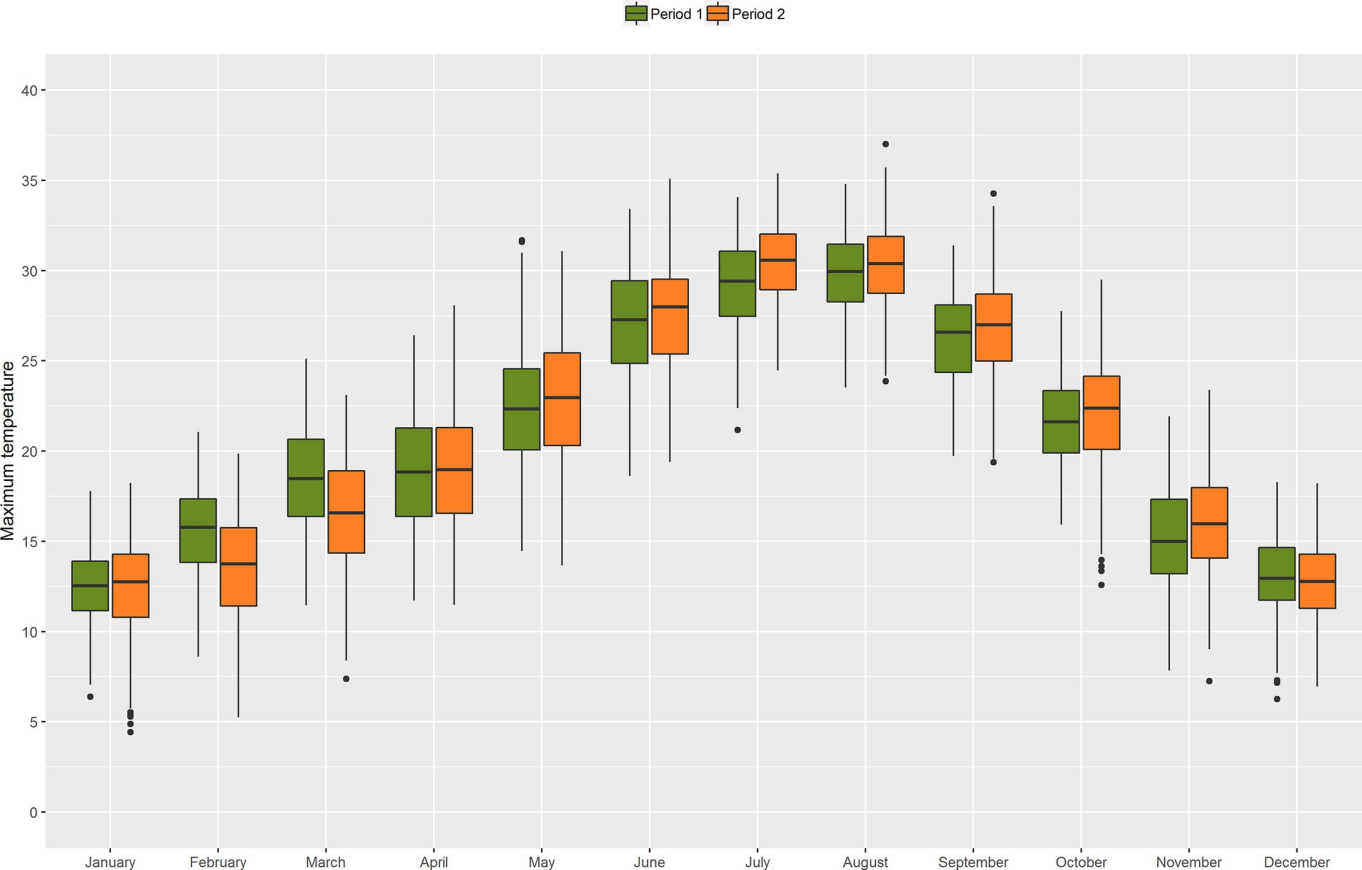
Maximum temperature distribution before (1993–2002) and after (2004–2013) the implementation of the Spanish Heat Health Prevention Plan.

When we conducted the analyses for the two periods, we observed that the average daily number of hospitalizations was higher in period 2 (period 1: 4,960; period 2: 6,611). For the three causes studied, a higher percentage of elderly patients were observed in period 2, following the aging of the Spanish population structure (Table 2). Period 2 recorded higher temperatures, mostly during the warm months (May-October), although winters in the period 2 were colder (January-March) (Fig 1). In the majority of the Spanish provinces period 2 was warmer, with a difference in daily mean temperature ranging from 0.1°C in areas like Burgos and Asturias to 1.0°C in Girona (S4 Table).

**Table 2:** Descriptive statistics on daily number of hospital admissions in Spain by causes, sex and age group for the two study periods (1997–2002 and 2004–2013).

	PERIOD 1 (1997–2002)					PERIOD 2 (2004–2013)				
	Total number of hospital admissions	% of hospital admissions	Daily hospital admissions			Total number of hospital admissions	% of hospital admissions	Daily hospital admissions		
			Mean	Min.	Max.			Mean	Min.	Max.
**TOTAL**	10,867,579		4,960.10	22	7,883	24,148,102		6,610.50	33	9,090
**CARDIOVASCULAR DISEASES**										
**Sex**										
Women	554,673	43.1	253.2	0	449	1,304,579	44.7	357.1	1	589
Men	733,022	56.9	334.6	3	590	1,614,279	55.3	441.9	4	681
Total	1,287,825		587.8	3	1,024	2,918,967		799.1	8	1,212
**Age group**										
16–64	361,022	28	164.8	1	286	701,424	24.0	192	1	289
65–74	385,125	29.9	175.8	1	327	646,360	22.1	176.9	0	332
75–84	384,802	29.9	175.6	1	330	1,026,178	35.2	280.9	1	472
> = 85	150,320	11.7	68.6	0	145	531,103	18.2	145.4	1	295
**CEREBROVASCULAR DISEASES**										
**Sex**										
Women	184,164	46.9	84.1	0	142	396,464	46.8	108.5	0	163
Men	208,314	53.1	95.1	0	154	450,219	53.2	123.2	0	178
Total	392,529		179.2	0	284	846,725		231.8	0	320
**Age group**										
16–64	88,602	22.6	40.4	0	82	190,192	22.5	52.1	0	87
65–74	116,223	29.6	53.0	0	96	191,586	22.6	52.4	0	93
75–84	133,458	34	60.9	0	105	308,817	36.5	84.5	0	131
> = 85	53,348	13.6	24.3	0	52	154,285	18.2	42.2	0	83
**RESPIRATORY DISEASES**										
**Sex**										
Women	445,062	35.0	203.1	0	685	1,228,758	38.4	336.4	0	902
Men	827,642	65.0	377.7	1	1,104	1,974,585	61.6	540.5	0	1,350
Total	1,272,949		581.0	2	1,789	3,203,568		877	2	2,245
**Age group**										
16–64	288,573	22.7	131.7	0	374	666,756	20.8	182.5	0	513
65–74	278,911	21.9	127.3	0	448	545,144	17.0	149.2	0	498
75–84	312,704	24.6	142.7	0	467	921,157	28.8	252.2	0	733
> = 85	141,247	11.1	64.5	0	227	566,274	17.7	155	0	540
**Number of inhabitants**^**a**^		39,852,651				46,727,890				
>65 years (%)		16.3				17.6				

^a^ The number of inhabitants was obtained from the Spanish National Institute for Statistics in 1997 (representing Period 1) and 2013 (representing Period 2). This data shows the population growth in Spain over the study period.

**Fig 2:**
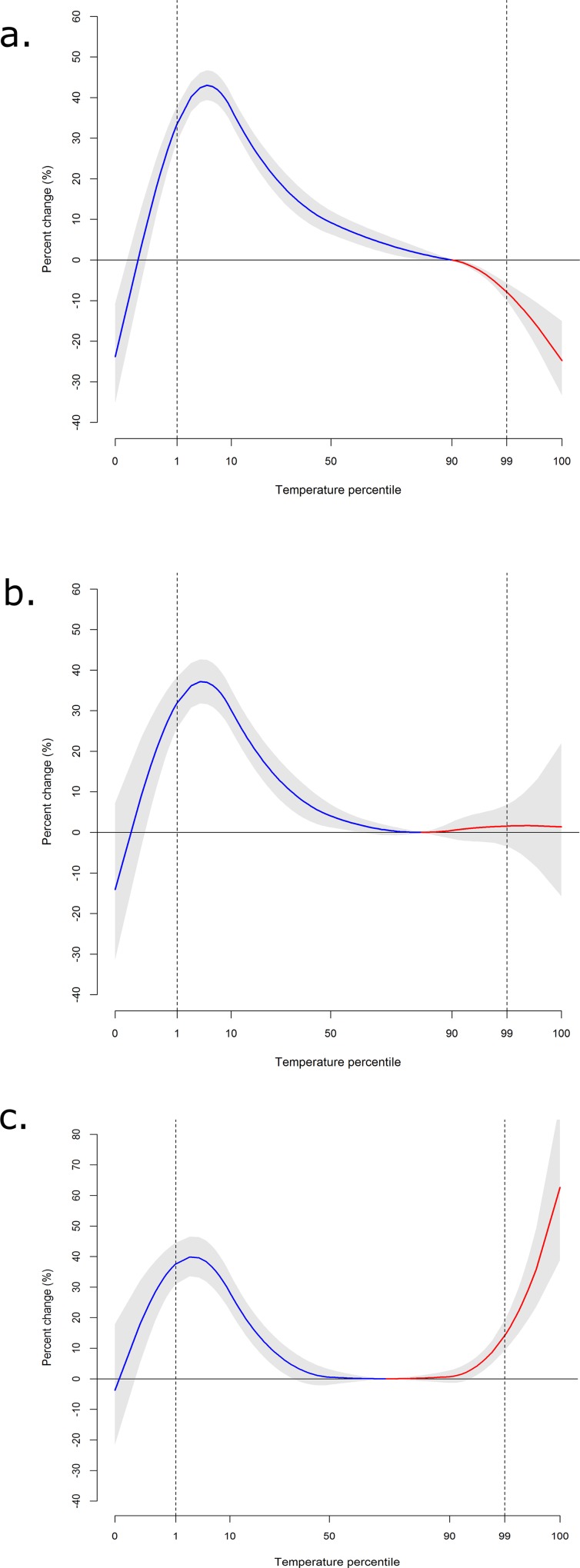
Overall cumulative exposure-response relationship (hospitalizations) in Spain for the period 1997–2013 and for different causes: cardiovascular diseases (2a), cerebrovascular diseases (2b) and respiratory diseases (2c).

Fig 2 represents the exposure-response curves of the relationship between maximum temperature and hospital admissions for cardiovascular (Fig 2A), cerebrovascular (Fig 2B) and respiratory (Fig 2C) diseases during the entire study period (1997–2013). The risk of hospitalization increased with cold temperatures for all the causes analysed, even though for temperatures below the 1st percentile, the upward trend was not observed. In contrast, the pattern for heat was heterogeneous for the three causes of hospitalization. While an increased risk of hospitalization with heat was observed for respiratory diseases (Fig 2C), heat decreased the risk of hospitalization for cardiovascular causes (Fig 2A). Regarding cerebrovascular diseases, heat was not significantly associated with the risk of hospitalization.

Table 3 reports the association for extreme cold and heat (1st and 99th percentiles of temperature, respectively) in comparison with the temperature of minimum risk (MHP). Cold effects were associated with a 34% (95% Confidence Interval (CI): 29%,38%) increase in hospitalizations for cardiovascular diseases. For heat, a protective effect was observed for cardiovascular hospital admissions (-8%, 95%CI: -10%,-6%). Cold increased the risk for cerebrovascular hospitalizations by 32% (95%CI: 26%,38%). Heat was not associated with the risk of hospital admissions for cerebrovascular diseases (2%, 95%CI: -3%,7%). For respiratory diseases, the risk of hospitalization significantly increased by 38% for cold (95%CI: 31%,45%). Heat was also associated with an increased risk of hospitalization for respiratory causes (14%, 95%CI: 9%,19%). Despite this, heat effects were much lower than cold effects. The effects of both cold and heat were higher among women for cardiovascular and cerebrovascular diseases, while for respiratory hospitalizations similar effects were observed for both sexes. Cold-related risks were high for all age groups, although a bit lower for those <65 years for cardiovascular and cerebrovascular diseases. For heat, the observed association with respiratory hospitalizations increased with age (Table 3).

**Table 3:** Percent change (%) and 95% confidence intervals for the relationship between cold and heat and hospitalizations in Spain for sex, age and cause of hospitalization for the period 1997–2013.

	Cold	Heat	MHP
**Cardiovascular diseases**			
**Total**	34 (29,38)*	-8 (-10,-6)*	**90**
Sex			
Women	41 (35,47)*	-9 (-12,-6)*	90
Men	27 (22,32)*	-8 (-10,-5)*	86
Age			
16–64	18 (13,24)*	-6 (-10,-1)*	79
65–74	29 (20,38)*	-10 (-13,-5)*	90
75–84	41 (34,49)*	-10 (-13,-7)*	90
> = 85	47 (37,56)*	-6 (-12,0)	90
**Cerebrovascular diseases**			
**Total**	32 (26,38)*	**2 (-3,7)**	**80**
Sex			
Women	43 (34,53)*	4 (-2,10)	90
Men	25 (16,35)*	0 (-7,9)	67
Age			
16–64	17 (8,28)*	4 (-6,15)	68
65–74	30 (18,43)*	0 (-8,9)	90
75–84	40 (31,50)*	-1 (-8,6)	75
> = 85	40 (26,55)*	4 (-5,14)	90
**Respiratory diseases**			
**Total**	38 (31,45)*	14 (9,19)*	**69**
Sex			
Women	38 (31,46)*	19 (11,28)*	57
Men	37 (31,45)*	11 (7,16)*	74
Age			
16–64	46 (37,56)*	5 (-2,13)	76
65–74	45 (33,58)*	12 (5,19)*	90
75–84	39 (30,48)*	17 (10,24)*	82
> = 85	48 (38,59)*	32 (20,46)*	48

MHP: Minimum Hospitalizations Percentile

Models for respiratory diseases were not control for influenza epidemics.

*p_value<0.05

Similar findings for cold were observed when we excluded from the analysis year 2003 (S5 Table and S1 Fig). However, the effects of heat on all-cause hospital admissions were slightly smaller after the exclusion of 2003. These results are consistent with the fact that temperatures registered on summer 2003 were exceptionally high, which modifies the effects estimates in hospitalizations.

**Fig 3:**
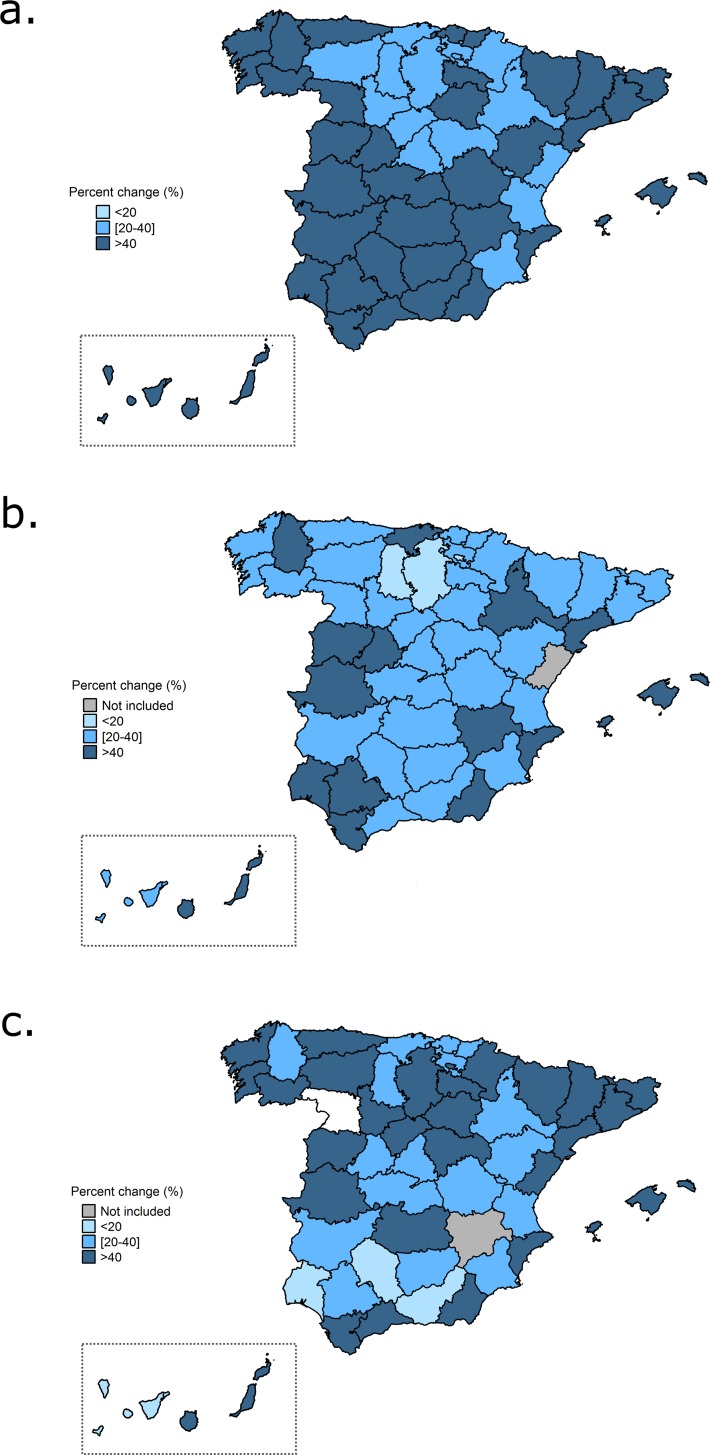
Percent change (%) for the relationship between cold and hospitalizations by Spanish provinces for the period 1997–2013 and different causes: cardiovascular diseases (3a), cerebrovascular diseases (3b) and respiratory diseases (3c).

**Fig 4:**
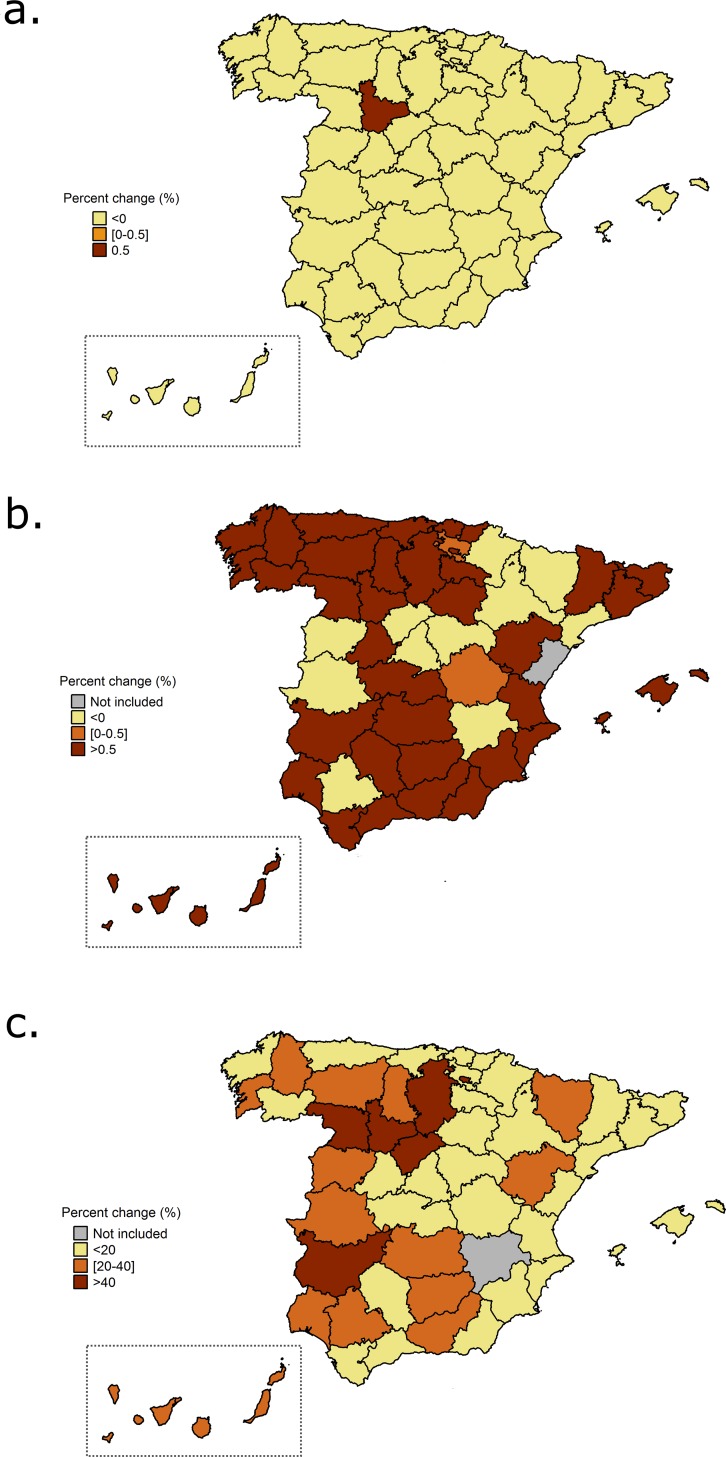
Percent change (%) for the relationship between heat and hospitalizations by Spanish provinces for the period 1997–2013 and different causes: cardiovascular diseases (4a), cerebrovascular diseases (4b) and respiratory diseases (4c).

The distribution of temperature-related hospitalizations for cold and heat by Spanish provinces is represented in Fig 3 and Fig 4, respectively. A different geographical pattern was observed depending on the cause of hospitalization. For cold, the risk of cardiovascular admissions was higher than 40% in all provinces, except in the center-north and some areas in the Mediterranean coast (Fig 3A). The risk of cerebrovascular admissions for cold temperatures was heterogeneously distributed around Spain, where the provinces with highest effects were located in the north, west and east (Fig 3B). For respiratory diseases, cold had the highest influence on hospitalizations in the north, but also in the east and west (Fig 3C). In general, as previously reported, lower effects for the exposure to heat were observed and also varied according to the cause of hospitalization. The protective effect seen for cardiovascular hospitalizations and heat was observed in all the provinces, except in one region, Valladolid (Fig 4A). More variability in the effects for heat and cerebrovascular hospitalizations were present among the Spanish provinces (Fig 4B). The majority of the provinces presented risks below 0, except some areas in the north-east, center of the country and south. Finally, we observed that heat had more impact on respiratory hospitalizations in the western provinces (Fig 4C). Note that the effects of heat on respiratory diseases were higher and therefore this map was represented using a different scale.

**Fig 5:**
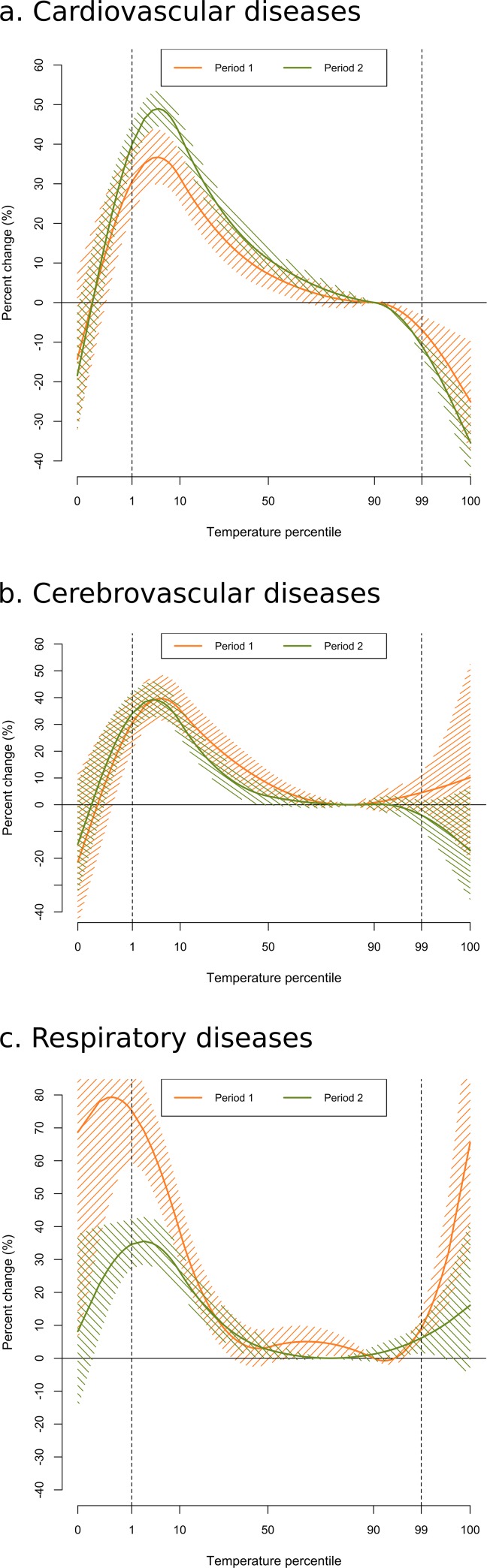
Overall cumulative exposure-response relationship (hospitalizations) for the two study periods (1997–2002 and 2004–2013) and for different causes: cardiovascular diseases (5a), cerebrovascular diseases (5b) and respiratory diseases (5c).

In terms of the analysis of temporal variations, Fig 5 shows the relationship between temperature and hospitalizations for the two study periods and for the three causes studied. When we compared the two study periods, the period-specific curves overlaid for cardiovascular and cerebrovascular admissions for both cold and heat (Fig 5A and 5B). However, period 2 showed a large reduction of the effects of cold for respiratory causes and a slight decline of heat (Fig 5C).

Cause-specific associations for extreme heat and cold for the two study periods were reported in Table 4. There was a non-significant increase in cold-related hospitalizations for cardiovascular in period 2 (period 1: 31%, 95%CI: 22%,39%; period 2: 40%, 95%CI: -35%,45%); however for heat a non-significant reduction was observed for cardiovascular hospitalizations (period 1: -7%, 95%CI: -10%,-3%; period 2: -11%, 95%CI: -13%,-8%). For cerebrovascular admissions, a small increase for cold was detected in period 2, even though this was not significant (period 1: 31%, 95%CI: 21%,34%; period 2: 34%, 95%CI: 26%,42%). Heat was protective for the risk of hospital admissions for cerebrovascular diseases mainly in period 2 (-23%, 95%CI: -29%,-16%), whereas it was a non-significant risk factor in period 1 (5%, 95%CI: -3%,12%). For respiratory diseases, the risk of cold-related hospitalization experienced a large reduction in period 2 (period 1: 75%, 95%CI: 60%,92%; period 2: 35%, 95%CI: 27%,42%). Heat slightly reduced the risk of respiratory hospitalizations in period 2 (period 1: 9%, 95%CI: 4%,14%; period 2: 6%, 95%CI: 2%,11%). Differences in the relationship between temperature and hospitalizations in the two periods were significant for all three causes (p<0.05). Cold-related hospitalizations for cardiovascular and cerebrovascular diseases increased in period 2 especially among men and the elderly. Nonetheless, heat-related hospitalizations for respiratory diseases showed a reduction for both sexes and all the age-groups, more remarkable for the elderly. An interesting finding is that in period 1 heat was significantly associated with respiratory admissions only among men, while this pattern was reversed in period 2, where only women presented a significant association (Table 4).

**Table 4:** Percent Change (%) and 95% Confidence Intervals for the relationship between cold and heat and mortality in Spain for the Period 1 (1997–2002) and Period 2 (2004–2013).

	PERIOD 1			PERIOD 2		
	COLD	HEAT	MHP	COLD	HEAT	MHP
**Cardiovascular diseases**						
**Total**	31 (22,39)*	-7 (-10,-3)*	**90**	40 (35,45)*	-11 (-13,-8)*	**90**
Sex						
Women	42 (31,53)*	-10 (-15,-5)*	90	45 (38,52)*	-13 (-16,-9)*	90
Men	24 (16,33)*	-3 (-9,2)	73	34 (28,41)*	-10 (-14,-7)*	
Age						
16–64	18 (9,27)*	0 (-9,8)	75	26 (19,34)*	-7 (-12,-1)*	82
65-74^(^^a^^)^	33 (19,48)*	-10 (-16,-4)*	90	36 (26,47)*	-12 (-17,-6)*	90
75-84^(^^b^^)^	38 (26,50)*	-9 (-15,-2)*	78	46 (37,54)*	-14 (-18,-10)*	90
> = 85^(^^c^^)^	37 (23,51)*	-9 (-19,3)	71	50 (39,61)*	-10 (-15,-4)*	90
**Cerebrovascular diseases**						
**Total**	31 (21,41)*	**5 (-3,12)**	**80**	34 (27,42)*	**-4 (-9,2)**	**82**
Sex						
Women	44 (25,66)*	11 (1,23)*	90	42 (31,54)*	-6 (-13,2)	90
Men	22 (11,34)*	-1 (-10,10)	70	31 (20,44)	-1 (-10,8)	66
Age						
16–64	21 (5,40)*	0 (-12,15)	79	13 (2,25)*	0 (-12,13)	62
65-74^(^^d^^)^	27 (7,50)*	1 (-11,14)	90	33 (17,52)*	-8 (-18,2)	79
75–84	39 (24,57)*	6 (-6,19)	72	42 (30,54)*	-4 (-12,5)	73
> = 85^(^^e^^)^	18 (-8,52)	1 (-18,24)	90	45 (29,64)*	-9 (-19,2)	88
**Respiratory diseases**						
**Total**^**(**^^**f**^^**)**^	75 (60,92)*	9 (4,14)*	**90**	35 (27,42)*	6 (2,11)*	**74**
Sex						
Women	72 (52,95)*	7 (-1,15)	90	37 (28,47)*	9 (2,17)*	73
Men	68 (53,85)*	7 (2,14)*	90	34 (26,42)*	4 (-1,9)	75
Age						
16–64	76 (53,103)*	6 (-3,16)	90	44 (34,55)*	-3 (-10,6)	77
65-74^(^^g^^)^	82 (62,105)*	6 (-4,17)	90	36 (25,48)*	5 (-2,14)	77
75-84^(^^h^^)^	101 (78,128)*	14 (5,25)*	90	33 (22,45)*	9 (2,16)*	90
> = 85	100 (74,129)*	26 (7,48)*	43	40 (29,53)*	21 (10,32)*	71

MHP: Minimum Hospitalizations Percentile

Models for respiratory diseases were not control for influenza epidemics. Some provinces were excluded from the model due to convergence problems.

(a) 1 province

(b) 1 province

(c) 1 province

(d) 4 provinces

e) 1 province

(f) 1 province

(g) 4 provinces

(h) 1 province

*p-value<0.05

Results from the analysis of temporal trends in temperature-related hospitalizations were robust to changes in the definition of the two study periods (S6 Table). However, when we considered two periods with similar number of years (period 1 from 1997–2004 and period 2 from 2005–2013), cold effects for respiratory hospitalizations were smaller in period 1, while no differences were observed in period 2.

Table 5 summarizes the risk associated with days in which the Spanish HHPP was activated (period 2) or would have been activated using the same criteria (period 1). The number of days the Spanish plan would have been activated according to the criteria outlined in the plan was higher in period 2 (period 1: 811 days, resulting in 116 days/year; period 2: 2,876 days, resulting in 288 days/year). High variability was detected according to the different Spanish provinces (S2 Fig and S7 Table), with the plan being activated more days in the South of Spain (e.g. Almeria, Ciudad Real, Malaga, Sevilla, Toledo). For cardiovascular diseases, a small and non-significant increased risk of hospitalization was observed in days in which the plan would have been activated in period 1 (1%, 95%CI: -2%,3%), whereas in period 2 the activation of the plan showed a small protective effect (-1%, 95%CI: -3%,0%). In terms of sex and age, we did not detect a clear pattern for cardiovascular hospitalizations. In period 1, the associations were similar in all age groups, but in period 2 those individuals aged 16–64 showed a significant reduction in risk during the activation of the plan. A protective effect of days of (potential) activation of the plan was observed for cerebrovascular diseases, which was slightly higher in period 2, although in none of the periods this was significant (period 1: -1%, 95%CI: -7%,5%; period 2: -2%, 95%CI: -4%,0%). No clear patterns were observed by age groups for cerebrovascular diseases. Respiratory-related hospitalizations increased by 5% (95%CI: 1%,9%) in days of potential activation of the in period 1, and by 4% (95%CI: 3%,6%) in days of activation of the plan in period 2, showing a small reduction in period 2. Higher risks were observed among women and the elderly, despite no association was found for women and those older than 85 in period 1.

**Table 5:** Percent Change (%) and 95% Confidence Intervals for the activation of the Spanish Heat Health Prevention Plan in the two study periods (1997–2002 and 2004–2013).

	PERIOD 1	PERIOD 2
**N° days activation HHPP**	811	2,876
**Cardiovascular diseases**		
**Total**	**1 (-2,3)**	**-1 (-3,0)**
Sex		
Women	-1 (-5,4)	-1 (-3,0)
Men	2 (-1,6)	-2(-3,0)
Age		
16–64	2 (-3,7)	1 (-1,3)
65–74	1 (-4,6)	-4 (-7,-2)*
75–84	2 (-5,9)	-1 (-3,0)
> = 85	1 (-7,5)^(^^a^^)^	-2 (-4,0)
**Cerebrovascular diseases**		
**Total**	**-1 (-7,5)**	**-2 (-4,0)**
Sex		
Women	7 (0,15)	-3 (-6,0)
Men	-3 (-9,3)	0 (-3,2)
Age		
16–64	-5 (-14,5)^(^^b^^)^	-1 (-5,2)
65–74	6 (-5,11)^(^^c^^)^	2 (-2,6)
75–84	4 (-4,13)	-3 (-6,0)
> = 85	-5 (-18,11)^(^^d^^)^	-3(-7,2)
**Respiratory diseases**		
**Total**	5 (1,9)*	4 (3,6)*
Sex		
Women	5 (-1,11)	5 (3,7)*
Men	4 (0,8)	4 (2,5)*
Age		
16–64	3 (-4,11)	0 (-3,2)
65–74	7 (0,14)	4 (1,7)*
75–84	8 (1,16)*	6 (3,8)*
> = 85	-2 (-11,7)	9 (5,13)*

Models for respiratory diseases were not control for influenza epidemics.

32 provinces were included in the model, in where the plan was activated in both periods. Some provinces were excluded from the model due to a high variability and unstable results:

(a) 1 province

(b) 1 province

(c) 1 province

(d) 2 provinces.

*p-value<0.05

Similar results were reported when we looked at different definitions of heat waves (S8 Table). The most remarkable result was the strong increase in the risk of hospital admission for respiratory causes with the longest and more intense heat waves (e.g. 38% (95% CI: 19,59) increase in periods of 4 or more consecutive days with temperatures above the 95th percentile) in period 1. Those increased risks were also found in period 2, but of smaller magnitude.

Results from sensitivity analyses showed some variations when modelling choices were tested (lag structure and degrees of freedom for day of season; S9 Table). In general, smaller effects were seen using shorter lag periods, while longer lag periods tended to reduce the effects on heat and increase the cold effects. Applying fewer degrees of freedom for seasonality showed in general higher effects for both cold and heat. However, when we selected more degrees of freedom we saw higher effects for cold and heat, except for heat and respiratory hospitalizations.

## 4. Discussion

We performed a country-wide study on the relationship between ambient temperature and hospital admissions in Spain. Our results showed an increase in the risk of hospital admissions for respiratory causes with heat. Heat did not increase the risk of hospitalizations by cardiovascular or cerebrovascular diseases. Cold temperatures increased the risk of cardiovascular, cerebrovascular and respiratory diseases. The study period (1997–2013) was divided in two, the second one characterized by the introduction of a HHPP. There was a slight reduction of heat-related hospitalizations for respiratory diseases in period 2, and slight increases in cold-related hospitalizations for cardiovascular and cerebrovascular diseases in period 2 compared to period 1. However cold-related hospitalizations for respiratory diseases showed an important drop in period 2 compared to period 1.

Our results showed that the number of cardiovascular and cerebrovascular hospital admissions decreases with heat, in contrast to mortality counts for the same causes [1,16]. Although intuitively one would expect that if heat increases the risk of mortality, the same would be observed with hospital admissions, this discrepancy has been reported before in several studies. For instance, different systematic reviews found no association with cardiovascular [16,17] and cerebrovascular morbidity [16]. In Greater London, United Kingdom, a 3.3% increase in all-cause mortality was observed for 1°C increase in temperature above 21.5°C, while there was no increase in all-cause emergency admissions [46]. The pattern of hospital admissions in Madrid was also different than that of mortality, with smaller heat effects for hospitalizations [23]. In Rhode Island, an increase in 1.3% of heat-related all emergency department visits was estimated, whereas for the same population and period, a 4.0% increase in heat-related mortality was reported [47]. In that study, however, the authors did not find any association for heat and emergency department visits for cardiovascular and respiratory causes. The differences between mortality and morbidity effects could be explained by the fact that many deaths from circulatory diseases occur rapidly in isolated people before they call for help and, therefore, before these patients receive medical treatment or are admitted to a hospital [48]. It should also be noted that those admitted to a hospital for one cause may die because of a different one. Thus, according to our and previous studies, cardiovascular and cerebrovascular hospital admissions are not a good metric to assess the health effects of heat, and therefore of the effectiveness of heat prevention plans.

In contrast, we found associations of heat with respiratory admissions. This also agrees with the literature. For example, Turner et al. analysed twelve studies that provide heat effects on respiratory hospital admissions [17]. They conducted a meta-analysis and reported an increase of 3.2% in respiratory morbidity with 1°C increase on hot days. Similar heat effects were reported for morbidity among the elderly [18]. One of the potential reasons for detecting heat effects with respiratory admissions is that respiratory events may not be as rapidly fatal as cardiovascular or cerebrovascular events [48]. Our results of the effects of respiratory admissions for different definitions of heat wave found the greatest risks for respiratory admissions in extremely hot and longer heat waves. These findings are similar to those reported elsewhere [14,49]. Thus, evaluations of the effectiveness of HHPPs could consider changes in respiratory hospital admissions as a relevant indicator. In the case of Spain, our evaluation detected only a small reduction during the days of activation of the plan, although the reduction was greater during the longest and intense heat waves. Bearing in mind that other alternative explanations are possible, as discussed below, this may suggest that the preventive plan had some beneficial effects on respiratory morbidity.

In contrast to heat, we detected that cold exposure led to significant increases in the risk of hospitalizations for cardiovascular, cerebrovascular and respiratory diseases. Results on the effect of cold on hospitalizations in the scientific literature are scarce and inconsistent. Our findings differ from that reported in a systematic review (including data from studies in countries like Italy, California, United Kingdom, Brazil or Hong Kong), in which the risk of morbidity outcomes reduced 0.28% and 0.46% for cardiovascular and cerebrovascular diseases, respectively, per 1°C decrease in temperature [18]. For respiratory diseases, that study found an increased risk of 2.70% in hospitalizations associated with cold temperatures. Wichmann et al. found no association between apparent temperature during the cold period and any of the cardiovascular, cerebrovascular and respiratory admissions [50]. Other authors have studied the effects of cold on more specific causes. For example, some studies examined specifically ACS or MI, common among cardiovascular disease patients. In this case, low temperatures were associated with ACS and MI in Greece [20], England and Wales [22] and California [51]. However, in Copenhagen and Hong Kong a protective effect of cold was found on ACS hospitalizations [6,8]. In Spain, in spite of having one of the milder winter climates in Europe, fuel poverty has been considered a health risk factor which increases the excess winter deaths [52]. This factor, as well as the ageing population, could explain these differences with our increase in respiratory admissions associated with low temperatures.

A difference in the temperature-related hospitalizations association was observed among regions in Spain. Whereas provinces in the south, west and north-east showed the highest cold effects for cardiovascular hospitalizations, provinces in the north presented higher risk for respiratory admissions at extreme cold although no clear pattern was seen for cerebrovascular diseases and cold. For heat, western areas presented the highest risks of respiratory admissions. These differences might be due to a variation in climate but also to other factors, such as differences in population distribution (the proportion of elderly), healthcare system and social services. Our results are in line with previous studies assessing the effect of heat on mortality in Spain [53,54]. In these studies, authors also found a geographic variation in the risk of mortality during summer months, also when considering the official thresholds used in the HHPP.

To the best of our knowledge, this is the first study assessing temporal variations in the temperature-hospitalizations association. So far, studies of temperature-related health trends have mainly focused on mortality. Results showed that in some countries there was a decline in mortality due to heat in the last decades, while for cold, a different pattern was observed depending on the region [30]. Our results comparing the two periods for the effects of cold led to different conclusions depending on the cause. We observed a significant decline for cold-related respiratory admissions in period 2, while the risk of cold-related hospitalizations for cardiovascular and cerebrovascular diseases increased in period 2. Even though there is no scientific evidence on the temporal changes of cold effects on morbidity, different recent studies in Spain have documented a decrease in cold-related mortality for all causes over the last decades [30], one of them using the same study period 2 [35]. Another study in Spain claimed for the need for a prevention plan for the effects of cold in Spain, which at the moment does not exist [55]. Indeed, Carmona et al. found that overall in Spain cold temperatures increase by 13% mortality due to natural causes, and by 18% and 24% for circulatory and respiratory causes. The increase in cold-related cardiovascular and cerebrovascular hospital admissions in the most recent period further justifies the need for such prevention plan. Some factors may influence changes we observed in the temperature-hospitalizations association, such as climate variability, the ageing of population, the prevalence of some diseases, fluctuations in the influenza vaccination coverage, as well as previous seasonal mortality.

As far as we know, our study is the first evaluating the effectiveness of a national HHPP focusing on morbidity. Only one study was found to evaluate a city-specific intervention for preventing morbidity effects of heat in Turin [56]. In this article, restricted to one year (2004) and to the elderly, the authors found a weak reduction in hospitalizations among the group that received a program based on soft home care services and an offer of social caretaking. So far, country-wide studies comparing the effectiveness of public interventions have shown decreases in heat-related mortality after the introduction of adaptation measures [33,57]. A systematic review, including articles from Europe, Canada, United States and Australia, concluded that the effects of heat on mortality reduced after the introduction of adaptive measures [33]. Recently, our group conducted a study assessing temporal changes in temperature-related mortality in Spain using the same period 2 (2004–2013) [35]. In this study, we found a small decrease in mortality attributable to extreme heat in the period after the implementation of the Spanish HHPP. A reduction was also observed for days of potential activation of the plan, using the thresholds established in the plan. In comparison, our present results with hospital admissions only found small reductions of respiratory hospitalizations after the implementation of the Spanish HHPP. Some differences between these two studies have to be mentioned, such as the number of years included in period 1, which was longer for mortality.

In this study, we used hospital admissions to assess temperature-related morbidity; however, other metrics have been used. We found that heat was not associated with cardiovascular and cerebrovascular hospitalizations. Similar findings were reported when using emergency department visits in USA [47]. By contrast, Liang et al. analysed emergency room visits and they estimated an increase of 15% for ACS [11]. In addition, high temperatures were also linked with daily emergency visits for acute MI, a common specific disease for cardiovascular patients [9], and with ambulance dispatches for respiratory diseases but not for cardiovascular diseases [21]. In the case of cold, we found higher hospitalization-risk for cardiovascular, cerebrovascular and respiratory diseases. Inconsistent results have been shown evaluating different outcomes. For instance, in the United Kingdom cold temperatures were associated with GP consultations for respiratory diseases but not for cardiovascular diseases among the elderly [15,58]. Nonetheless, cold temperatures increased cardiovascular disease emergency room visits in Canada [59] and emergency visits for MI in Korea [9].

The results of this study have several implications. Climate change has been considered one of the major threats for human health [60]. As the Mediterranean region will suffer above average rising temperatures, there is an urgent need to introduce public interventions to reduce the health effects of extreme temperatures. Until now, the majority of the evaluations of HHPPs have focused on reducing mortality. However, other impacts should be considered. We observed that heat significantly increased respiratory hospitalizations; therefore heat prevention plans should include this health indicator to better target preventive measures and the most affected populations. In addition, despite the majority of the HHPP, also the Spanish one, have been designed considering thresholds based on the increase in temperature-related mortality, the temperature in which respiratory admissions starts rising should also be included in these early warning systems. Moreover, prevention measures should focus on the most vulnerable populations, including isolated people with less social contact, as it has been hypothesized that circulatory diseases occur rapidly and fatal before the patient goes to hospital. Another aspect to consider is the fact that, in contrast to mortality, we found no heat association with cardiovascular and cerebrovascular hospitalizations. Consequently, more research is needed in order to understand the effects of heat on cardiovascular and cerebrovascular patients, probably considering mortality and hospitalizations together to account for competing risks. Additionally, as mentioned above, our results support that the Spanish prevention plan should also cover cold temperatures, but also the range of moderate heat and cold. It is important to note that, for temperatures below the 1st percentile, i.e. extreme cold, our curves showed reductions in risks compared to moderate cold. One hypothesis that could explain this finding is that people’s behaviour may change when extremely low temperatures are registered. That is, in those extremely cold days people could take more preventive measures, such as avoiding displacement and therefore spend more time inside their homes. However, further research is required to understand these results.

Different limitations in this study should be mentioned. First, we used maximum temperature registered from a single monitoring station in each province (located in the province capital). However, as we previously reported in a subanalysis with 4 provinces, temperature from different monitoring stations in a province had correlations above 0.87 [61]. Second, we split the study period into two (1997–2002 and 2004–2013) in order to compare temperature-related hospitalizations and evaluate the effectiveness of the Spanish HHPP. Period 1 only accounted for five years, while period 2 had ten years data. The shorter period 1 could be one of the reasons for not detecting differences between periods. However, we included hospitalizations since the beginning of the national register, which accounts for 92% of the admissions registered in Spain. Third, using hospital admissions as the outcome we were only able to detect the most severe cases of temperature-related morbidity, and less severe morbidity effects of temperatures were not captured in our study. Fourth, we did not include air pollution data because it was not available. Despite this, air pollutants have been described to be mediators between temperature and health outcomes rather than confounders [62]. Fifth, we performed a before-after comparison to assess temporal changes, however different approaches could be considered, such as the difference-in-difference approach [57] or interrupted time series (ITS) analysis, which establishes an underlying trend, ‘interrupted’ by the intervention at a specific point in time [63]. In an ideal ITS study, a high number of points are require to analyse specifically the trend. In our study we would have a limited number of (short) periods to compare the temporal trend. Moreover, if the trend was not lineal, using this approach we would not be able to attribute possible changes to the intervention. Nevertheless, the analysis we did in this study could be seen as an approximation of the ITS methodology, but instead of comparing temporal trends with several periods, we did include only two broader periods. Sixth, the design of our study (a before-after comparison) does not allow establishing causality. We found reductions in both heat and cold-related respiratory admissions, even though the Spanish HHPP only works for heat and there was not a specific cold preventive plan. Therefore, apart from the introduction of the HHPP, this decline could be explained for many other factors occurred in the second study-period, such as an improvement of the respiratory disease treatments, biological adaptation (changes in susceptibility) or demographic and socio-economic changes [33].

In conclusion, our study reported significant cold effects for cardiovascular, cerebrovascular and respiratory hospitalizations. However, we did not observe any heat association with cardiovascular and cerebrovascular hospitalizations. We also found a small decrease in heat-related respiratory hospitalizations in the period after the HHPP was introduced. In general, cold-related hospitalizations did not reduce during the study period, except for respiratory diseases, for which a significant decrease was reported in period 2. The elderly were more vulnerable to extreme temperatures. The inclusion of morbidity effects on weather-related prevention plans might reduce its impact on human’s health. Understanding the mechanisms of cold and heat-related hospitalizations is important to better protect public health, particularly among the most vulnerable populations.

## Supporting information

S1 Table Descriptive statistics on daily maximum temperature by year and period (period 1: 1997–200 and period 2: 2004–2013). (DOCX)

S2 Table Descriptive statistics on daily number of hospitalizations and daily maximum temperature by Spanish provinces (1997–2013). (DOCX)

S3 Table Descriptive statistics on daily number of all-cause hospital admissions and daily maximum temperature by month and day of the week (1997–2013). (DOCX)

S4 Table Descriptive statistics on daily number of hospitalizations and daily maximum temperature by Spanish provinces for the two study periods (1997–2002 and 2004–2013). (DOCX)

S5 Table Percent change (%) and 95% confidence intervals for the relationship between cold and heat and hospitalizations in Spain for sex, age and cause of hospitalization for the period 1997–2013, excluding year 2003. MHP: Minimum Hospitalizations Percentile. Models for respiratory diseases were not control for influenza epidemics. *p_value<0.05.
(DOCX)

S6 Table Percent Change (%) and 95% Confidence Intervals for the relationship between cold and heat and mortality in Spain for 1997–2004 (Period 1) and 2005–2013 (Period 2). MHP: Minimum Hospitalizations Percentile. Models for respiratory diseases were not control for influenza epidemics. Some provinces were excluded from the model due to convergence problems. (a) 1 province; (b) 1 province; (c) 1 province; (d) 4 provinces; (e) 1 province; (f) 1 province; (g) 4 provinces; (h) 1 province. *p-value<0.05.
(DOCX)

S7 Table Number of days in which the Spanish Heat Health Prevention Plan was activated and number of days considered heat wave, according to the different definitions, by periods (1997–2002 and 2004–2013) and Spanish provinces. Activation HHPP: thresholds established in each province to activate the Spanish Heat Health Prevention Plan.
(DOCX)

S8 Table Percent Change (%) and 95% Confidence Intervals for different definitions of heat waves in the two study periods (1997–2002 and 2004–2013). Some provinces were excluded from the model due to a high variability and unstable results: (a) 1 province; (b) 1 province, (c) 1 province, (d) 1 province. *p-value<0.05.
(DOCX)

S9 Table Sensitivity analyses. Df: degrees of freedom. *p-value<0.05.
(DOCX)

S1 Fig Overall cumulative exposure-response relationship (hospitalizations) in Spain for the period 1997–2013, excluding year 2003, and for different causes: cardiovascular diseases (a), cerebrovascular diseases (b) and respiratory diseases (c).
(TIF)

S2 Fig Number of days for the activation of the Spanish Heat Health Prevention Plan by provinces in the two study periods: a) period 1 (1997–2002) and b) period 2 (2004–2013).
(TIF)
