# Supplementary material for: Temporal changes in the effects of ambient temperatures on hospital admissions in Spain
Source: PLoS One. 2019 Jun 13;14(6):e0218262. doi: 10.1371/journal.pone.0218262 (PMC6564013; doi:10.1371/journal.pone.0218262)
Supplement: S1 Table — (DOCX) [file pone.0218262.s001.docx]

# S1 Table: Descriptive statistics on daily maximum temperature by year and period (period 1: 1997-200 and period 2: 2004-2013).

| **Year** | | **Daily Maximum Temperature** | | |
| --- | --- | --- | --- | --- |
|  |  | **Mean** | **Min** | **Max** |
| Period 1 | 1997 | 21.2 | 6.4 | 32.7 |
|  | 1998 | 20.9 | 7.8 | 34.8 |
|  | 1999 | 20.6 | 7.3 | 34.1 |
|  | 2000 | 20.8 | 8.2 | 33.9 |
|  | 2001 | 20.9 | 6.2 | 34.0 |
|  | 2002 | 20.8 | 10.2 | 32.8 |
|  | 2003 | 21.3 | 6.5 | 36.7 |
| Period 2 | 2004 | 20.7 | 7.7 | 35.4 |
|  | 2005 | 20.9 | 5.2 | 34.6 |
|  | 2006 | 21.4 | 5.8 | 35.3 |
|  | 2007 | 20.7 | 6.0 | 35.2 |
|  | 2008 | 20.5 | 8.1 | 33.9 |
|  | 2009 | 21.3 | 4.9 | 34.2 |
|  | 2010 | 20.2 | 4.4 | 35.0 |
|  | 2011 | 21.6 | 7.7 | 35.7 |
|  | 2012 | 21.1 | 6.2 | 37.0 |
|  | 2013 | 20.6 | 8.4 | 34.5 |
