# Supplementary material for: Temporal changes in the effects of ambient temperatures on hospital admissions in Spain
Source: PLoS One. 2019 Jun 13;14(6):e0218262. doi: 10.1371/journal.pone.0218262 (PMC6564013; doi:10.1371/journal.pone.0218262)
Supplement: S2 Table — (DOCX) [file pone.0218262.s002.docx]

# S2 Table: Descriptive statistics on daily number of hospitalizations and daily maximum temperature by Spanish provinces (1997-2013).

| **Province** |  | **Total number of hospitalizations** | **% of hospitalizations** |  | **Daily hospital admissions** | | |  | **Daily maximum temperature** | | |
| --- | --- | --- | --- | --- | --- | --- | --- | --- | --- | --- | --- |
|  |  |  |  |  | **Mean** | **Min** | **Max** |  | **Mean** | **Min** | **Max** |
| Alava |  | 337,662 | 0.9 |  | 54.4 | 0 | 107 |  | 17.5 | -1.5 | 40.8 |
| Albacete |  | 372,486 | 1 |  | 60 | 0 | 117 |  | 21.2 | -4.4 | 42 |
| Alicante |  | 1,526,308 | 4.1 |  | 245.8 | 0 | 457 |  | 23.5 | 6.5 | 40.4 |
| Almeria |  | 532,320 | 1.4 |  | 85.7 | 0 | 174 |  | 23.2 | 8.3 | 40.6 |
| Avila |  | 160,113 | 0.4 |  | 25.8 | 0 | 60 |  | 17.3 | -3.7 | 37.4 |
| Badajoz |  | 663,607 | 1.8 |  | 106.9 | 0 | 225 |  | 24 | 4 | 44.8 |
| Illes Balears |  | 662,724 | 1.8 |  | 106.7 | 0 | 229 |  | 22.7 | 4.8 | 41.4 |
| Barcelona |  | 4,265,539 | 11.4 |  | 687 | 0 | 1130 |  | 20.7 | 2.7 | 37.4 |
| Burgos |  | 451,596 | 1.2 |  | 72.7 | 0 | 137 |  | 17 | -2.5 | 38.8 |
| Caceres |  | 406,015 | 1.1 |  | 65.4 | 0 | 116 |  | 22.2 | 3.2 | 42.6 |
| Cadiz |  | 828,902 | 2.2 |  | 133.5 | 0 | 259 |  | 21.9 | 7.6 | 38 |
| Castellon |  | 438,921 | 1.2 |  | 70.7 | 0 | 147 |  | 22.7 | 6.4 | 40.6 |
| Ciudad Real |  | 559,672 | 1.5 |  | 90.1 | 0 | 154 |  | 22 | -0.2 | 42.5 |
| Cordoba |  | 579,210 | 1.6 |  | 93.3 | 0 | 182 |  | 25.3 | 3.8 | 46.2 |
| A Coruna |  | 1,025,219 | 2.8 |  | 165.1 | 0 | 270 |  | 18.3 | 4.6 | 34.5 |
| Cuenca |  | 235,285 | 0.6 |  | 37.9 | 0 | 77 |  | 19.8 | -0.1 | 39.7 |
| Girona |  | 573,881 | 1.5 |  | 92.4 | 0 | 160 |  | 21.6 | 2.9 | 41.2 |
| Granada |  | 722,970 | 1.9 |  | 116.4 | 0 | 219 |  | 23.3 | 1.1 | 42 |
| Guadalajara |  | 193,071 | 0.5 |  | 31.1 | 0 | 74 |  | 21.2 | 0.1 | 43.5 |
| Guipúzcoa |  | 695,983 | 1.9 |  | 112.1 | 0 | 212 |  | 19.3 | -1 | 39.8 |
| Huelva |  | 415,236 | 1.1 |  | 66.9 | 0 | 118 |  | 24.2 | 5 | 43.8 |
| Huesca |  | 234,440 | 0.6 |  | 37.8 | 0 | 74 |  | 20.1 | -4.4 | 41.4 |
| Jaen |  | 620,162 | 1.7 |  | 99.9 | 0 | 183 |  | 21.7 | 1.3 | 42.8 |
| Leon |  | 498,541 | 1.3 |  | 80.3 | 0 | 138 |  | 16.9 | -3 | 36.2 |
| Lleida |  | 351,821 | 0.9 |  | 56.7 | 0 | 109 |  | 21.7 | -5.8 | 40.8 |
| La Rioja |  | 289,878 | 0.8 |  | 46.7 | 0 | 91 |  | 19.9 | -3.5 | 40.6 |
| Lugo |  | 414,426 | 1.1 |  | 66.7 | 0 | 122 |  | 17.8 | -1.4 | 39.1 |
| Madrid |  | 4,319,542 | 11.6 |  | 695.7 | 0 | 1135 |  | 21.3 | 0.5 | 40.9 |
| Malaga |  | 994,542 | 2.7 |  | 160.2 | 0 | 278 |  | 23.6 | 6.8 | 42 |
| Murcia |  | 1,254,066 | 3.4 |  | 202 | 0 | 588 |  | 25.1 | 5.5 | 45 |
| Navarra |  | 561,101 | 1.5 |  | 90.4 | 0 | 149 |  | 18.2 | -2.1 | 40.6 |
| Ourense |  | 416,161 | 1.1 |  | 67 | 0 | 129 |  | 21.8 | 2.4 | 42 |
| Asturias |  | 1,208,514 | 3.2 |  | 194.6 | 0 | 348 |  | 17.2 | 3.7 | 36 |
| Palencia |  | 213,534 | 0.6 |  | 34.4 | 0 | 79 |  | 17 | -2.5 | 38.8 |
| Las Palmas |  | 561,297 | 1.5 |  | 90.4 | 0 | 168 |  | 24.4 | 15.8 | 39 |
| Pontevedra |  | 885,918 | 2.4 |  | 142.7 | 0 | 242 |  | 19.2 | 4.5 | 39.5 |
| Salamanca |  | 343,105 | 0.9 |  | 55.3 | 0 | 115 |  | 19.6 | -3 | 39.7 |
| Santa Cruz de Tenerife |  | 497,709 | 1.3 |  | 80.2 | 0 | 154 |  | 24.9 | 15.4 | 42.9 |
| Cantabria |  | 480,077 | 1.3 |  | 77.3 | 0 | 139 |  | 18.8 | 4 | 37.8 |
| Segovia |  | 144,067 | 0.4 |  | 23.2 | 0 | 49 |  | 18.1 | -2.6 | 38.3 |
| Sevilla |  | 1,240,350 | 3.3 |  | 199.8 | 0 | 330 |  | 25.7 | 4 | 45.9 |
| Soria |  | 97,272 | 0.3 |  | 15.7 | 0 | 47 |  | 17.5 | -4 | 36.8 |
| Tarragona |  | 639,824 | 1.7 |  | 103 | 0 | 195 |  | 21.5 | 2.2 | 38 |
| Teruel |  | 139,339 | 0.4 |  | 22.4 | 0 | 48 |  | 19.8 | -4.8 | 40.2 |
| Toledo |  | 566,724 | 1.5 |  | 91.3 | 0 | 161 |  | 22.4 | -0.4 | 43.1 |
| Valencia |  | 2,053,653 | 5.5 |  | 330.8 | 0 | 545 |  | 23.2 | 3.9 | 42.8 |
| Valladolid |  | 530,250 | 1.4 |  | 85.4 | 0 | 169 |  | 18.9 | -2 | 39.5 |
| Vizcaya |  | 1,012,892 | 2.7 |  | 163.1 | 0 | 285 |  | 19.5 | 2.1 | 41.9 |
| Zamora |  | 218,504 | 0.6 |  | 35.2 | 0 | 75 |  | 19.3 | -2.6 | 39.2 |
| Zaragoza |  | 844,492 | 2.3 |  | 136 | 0 | 233 |  | 21.5 | -3 | 43.1 |
