# Supplementary material for: Temporal changes in the effects of ambient temperatures on hospital admissions in Spain
Source: PLoS One. 2019 Jun 13;14(6):e0218262. doi: 10.1371/journal.pone.0218262 (PMC6564013; doi:10.1371/journal.pone.0218262)
Supplement: S3 Table — (DOCX) [file pone.0218262.s003.docx]

# S3 Table: Descriptive statistics on daily number of all-cause hospital admissions and daily maximum temperature by month and day of the week (1997-2013).

|  | **Daily hospital admissions** | | | |  | **Daily maximum temperature** | | |
| --- | --- | --- | --- | --- | --- | --- | --- | --- |
|  | **Mean** | **Min** | **Max** | **Total number** |  | **Mean** | **Min** | **Max** |
| **Month** |  |  |  |  |  |  |  |  |
| January | 6,611.50 | 2,671 | 9,090 | 3,484,260 |  | 12.5 | 4.4 | 18.2 |
| February | 6,540.20 | 2,663 | 9,022 | 3,139,291 |  | 14.1 | 5.2 | 21.1 |
| March | 6,330.70 | 2,551 | 8,265 | 3,336,260 |  | 17.3 | 7.4 | 25.1 |
| April | 6,157.40 | 2,496 | 8,126 | 3,140,271 |  | 19.1 | 11.5 | 28.1 |
| May | 6,097.00 | 2,471 | 7,893 | 3,213,113 |  | 22.7 | 13.7 | 31.7 |
| June | 5,996.40 | 2,335 | 7,854 | 3,058,148 |  | 27.5 | 18.6 | 35.1 |
| July | 5,814.70 | 2,255 | 7,596 | 3,064,339 |  | 30 | 21.2 | 35.4 |
| August | 5,686.50 | 2,225 | 7,348 | 2,996,768 |  | 30.3 | 23.5 | 37 |
| September | 5,857.40 | 2,274 | 7,574 | 2,987,295 |  | 26.6 | 19.4 | 34.3 |
| October | 6,063.40 | 2,327 | 8,031 | 3,195,435 |  | 21.8 | 12.6 | 29.5 |
| November | 6,098.20 | 2,303 | 7,999 | 3,110,092 |  | 15.7 | 7.3 | 23.4 |
| December | 4,845.60 | 22 | 7,775 | 2,553,649 |  | 12.8 | 6.2 | 18.3 |
|  |  |  |  |  |  |  |  |  |
| **Day of the week** | |  |  |  |  |  |  |  |
| Sunday | 4,911.20 | 33 | 7,287 | 4,356,240 |  | 20.9 | 4.4 | 36.2 |
| Monday | 6,731.40 | 39 | 9,090 | 5,970,713 |  | 20.8 | 5.2 | 35.9 |
| Tuesday | 6,531.60 | 54 | 8,725 | 5,793,566 |  | 20.9 | 7.3 | 36.7 |
| Wednesday | 6,377.50 | 22 | 8,581 | 5,656,835 |  | 20.9 | 5.5 | 36.1 |
| Thursday | 6,292.00 | 32 | 8,750 | 5,580,963 |  | 21 | 4.9 | 35.7 |
| Friday | 6,193.10 | 37 | 8,793 | 5,493,237 |  | 21 | 5.3 | 37 |
| Saturday | 4,991.40 | 38 | 7,212 | 4,427,367 |  | 20.9 | 5.4 | 35.7 |
