# Supplementary material for: Temporal changes in the effects of ambient temperatures on hospital admissions in Spain
Source: PLoS One. 2019 Jun 13;14(6):e0218262. doi: 10.1371/journal.pone.0218262 (PMC6564013; doi:10.1371/journal.pone.0218262)
Supplement: S4 Table — (DOCX) [file pone.0218262.s004.docx]

# S4 Table: Descriptive statistics on daily number of hospitalizations and daily maximum temperature by Spanish provinces for the two study periods (1997-2002 and 2004-2013).

|  | **PERIOD 1 (1997-2002)** | | | | | | | |  |  | **PERIOD 2 (2004-2013)** | | | | | | | | |
| --- | --- | --- | --- | --- | --- | --- | --- | --- | --- | --- | --- | --- | --- | --- | --- | --- | --- | --- | --- |
| **Province** | **Total number of hospital admissions** | **% of all hospital admissions** | **Number of hospital admissions per day** | | |  | **Daily maximum temperature** | | |  | **Total number of hospital admissions** | **% of all hospital admissions** | **Number of hospital admissions per day** | | |  | **Daily maximum temperature** | | |
|  |  |  | **Mean** | **Min** | **Max** |  | **Mean** | **Min** | **Max** |  |  |  | **Mean** | **Min** | **Max** |  | **Mean** | **Min** | **Max** |
| Alava | 106,522 | 0.98 | 48.6 | 0 | 101 |  | 17.6 | -0.1 | 38.7 |  | 212,242 | 0.88 | 58.1 | 0 | 107 |  | 17.4 | -1.5 | 40.8 |
| Albacete | 103,252 | 0.95 | 47.1 | 0 | 105 |  | 21.4 | 2.0 | 40.6 |  | 244,783 | 1.01 | 67.0 | 0 | 117 |  | 21.1 | -4.4 | 42.0 |
| Alicante | 392,928 | 3.62 | 179.3 | 0 | 323 |  | 23.6 | 9.4 | 38.2 |  | 1,045,680 | 4.33 | 286.3 | 1 | 457 |  | 23.5 | 6.5 | 40.4 |
| Almeria | 149,035 | 1.37 | 68 | 0 | 134 |  | 23.4 | 11.6 | 40.6 |  | 348,893 | 1.44 | 95.5 | 0 | 174 |  | 23.0 | 8.3 | 40.3 |
| Avila | 40,211 | 0.37 | 18.4 | 0 | 51 |  | 17.1 | -1.4 | 35.4 |  | 110,506 | 0.46 | 30.3 | 0 | 60 |  | 17.3 | -3.7 | 37.4 |
| Badajoz | 177,288 | 1.63 | 80.9 | 0 | 183 |  | 23.9 | 7.0 | 43.0 |  | 441,465 | 1.83 | 120.8 | 0 | 225 |  | 24.1 | 4.0 | 43.4 |
| Illes Balears | 121,817 | 1.12 | 55.6 | 0 | 147 |  | 23.0 | 7.2 | 41.4 |  | 493,141 | 2.04 | 135.0 | 0 | 229 |  | 22.5 | 4.8 | 38.7 |
| Barcelona | 1,255,643 | 11.55 | 573.1 | 0 | 1048 |  | 20.3 | 3.4 | 34.0 |  | 2,750,856 | 11.39 | 753.0 | 0 | 1130 |  | 20.9 | 2.7 | 37.4 |
| Burgos | 129,933 | 1.2 | 59.3 | 0 | 113 |  | 17.2 | -1.4 | 37.3 |  | 296,381 | 1.23 | 81.1 | 0 | 137 |  | 16.8 | -2.5 | 37.2 |
| Caceres | 133,476 | 1.23 | 60.9 | 0 | 106 |  | 22.0 | 4.6 | 41.0 |  | 250,768 | 1.04 | 68.6 | 0 | 116 |  | 22.3 | 3.2 | 42.6 |
| Cadiz | 262,657 | 2.42 | 119.9 | 0 | 259 |  | 21.9 | 11.0 | 36.0 |  | 514,355 | 2.13 | 140.8 | 0 | 220 |  | 22.0 | 7.6 | 38.0 |
| Castellon | 109,800 | 1.01 | 50.1 | 0 | 104 |  | 22.7 | 6.4 | 36.6 |  | 300,685 | 1.25 | 82.3 | 0 | 147 |  | 22.6 | 6.8 | 40.6 |
| Ciudad Real | 176,915 | 1.63 | 80.7 | 0 | 140 |  | 22.1 | 0.8 | 41.4 |  | 349,647 | 1.45 | 95.7 | 0 | 154 |  | 22.0 | -0.2 | 42.5 |
| Cordoba | 184,863 | 1.7 | 84.4 | 0 | 182 |  | 25.1 | 8.6 | 43.8 |  | 356,522 | 1.48 | 97.6 | 0 | 164 |  | 25.4 | 3.8 | 45.5 |
| A Coruna | 323,284 | 2.97 | 147.6 | 0 | 232 |  | 18.1 | 6.4 | 34.4 |  | 639,343 | 2.65 | 175.0 | 0 | 270 |  | 18.3 | 4.6 | 34.5 |
| Cuenca | 72,332 | 0.67 | 33 | 0 | 67 |  | 19.4 | 0.1 | 36.5 |  | 148,998 | 0.62 | 40.8 | 0 | 77 |  | 20.0 | -0.1 | 39.7 |
| Girona | 172,804 | 1.59 | 78.9 | 0 | 144 |  | 21.2 | 3.6 | 37.6 |  | 368,068 | 1.52 | 100.8 | 0 | 160 |  | 21.7 | 2.9 | 40.2 |
| Granada | 235,653 | 2.17 | 107.6 | 0 | 219 |  | 23.5 | 5.0 | 41.4 |  | 442,177 | 1.83 | 121.0 | 0 | 213 |  | 23.1 | 1.1 | 42.0 |
| Guadalajara | 50,007 | 0.46 | 22.8 | 0 | 52 |  | 21.1 | 1.0 | 40.0 |  | 131,809 | 0.55 | 36.1 | 0 | 74 |  | 21.1 | 0.1 | 43.5 |
| Guipuzcoa | 224,459 | 2.07 | 102.4 | 0 | 212 |  | 19.8 | 2.8 | 38.8 |  | 431,288 | 1.79 | 118.1 | 0 | 197 |  | 18.9 | -1.0 | 39.2 |
| Huelva | 132,162 | 1.22 | 60.3 | 0 | 118 |  | 23.9 | 8.8 | 40.2 |  | 256,011 | 1.06 | 70.1 | 0 | 118 |  | 24.3 | 5.0 | 43.8 |
| Huesca | 71,782 | 0.66 | 32.8 | 0 | 68 |  | 19.9 | -4.4 | 38.2 |  | 147,971 | 0.61 | 40.5 | 0 | 74 |  | 20.2 | -3.2 | 41.4 |
| Jaen | 199,925 | 1.84 | 91.2 | 0 | 183 |  | 21.6 | 5.0 | 39.4 |  | 378,118 | 1.57 | 103.5 | 0 | 167 |  | 21.8 | 1.3 | 42.8 |
| Leon | 158,643 | 1.46 | 72.4 | 0 | 130 |  | 16.7 | -1.6 | 35.8 |  | 309,884 | 1.28 | 84.8 | 0 | 138 |  | 16.9 | -3.0 | 36.2 |
| Lleida | 100,735 | 0.93 | 46 | 0 | 103 |  | 21.8 | -5.8 | 39.5 |  | 229,489 | 0.95 | 62.8 | 0 | 109 |  | 21.6 | -2.0 | 40.8 |
| La Rioja | 82,680 | 0.76 | 37.7 | 0 | 76 |  | 19.8 | 0.6 | 40.6 |  | 190,357 | 0.79 | 52.1 | 0 | 91 |  | 19.9 | -3.5 | 40.6 |
| Lugo | 135,306 | 1.25 | 61.8 | 0 | 106 |  | 17.6 | -1.4 | 36.2 |  | 254,485 | 1.05 | 69.7 | 0 | 122 |  | 17.9 | 0.5 | 37.7 |
| Madrid | 1,239,111 | 11.4 | 565.5 | 1 | 893 |  | 21.0 | 1.4 | 39.2 |  | 2,842,114 | 11.77 | 778.0 | 0 | 1135 |  | 21.5 | 0.5 | 40.9 |
| Malaga | 267,839 | 2.46 | 122.2 | 0 | 252 |  | 23.6 | 9.6 | 41.0 |  | 660,941 | 2.74 | 180.9 | 0 | 278 |  | 23.6 | 6.8 | 42.0 |
| Murcia | 347,961 | 3.2 | 158.8 | 0 | 310 |  | 25.1 | 7.6 | 41.8 |  | 832,581 | 3.45 | 227.9 | 0 | 588 |  | 25.1 | 5.5 | 45.0 |
| Navarra | 176,325 | 1.62 | 80.5 | 0 | 140 |  | 18.0 | 0.5 | 39.0 |  | 351,298 | 1.45 | 96.2 | 0 | 149 |  | 18.2 | -2.1 | 40.6 |
| Ourense | 124,710 | 1.15 | 56.9 | 0 | 110 |  | 21.5 | 3.6 | 40.6 |  | 263,921 | 1.09 | 72.2 | 0 | 129 |  | 22.0 | 2.4 | 41.6 |
| Asturias | 371,690 | 3.42 | 169.6 | 0 | 315 |  | 17.5 | 6.0 | 36.0 |  | 760,636 | 3.15 | 208.2 | 0 | 348 |  | 16.9 | 3.7 | 33.0 |
| Palencia | 65,135 | 0.6 | 29.7 | 0 | 62 |  | 17.2 | -1.4 | 37.3 |  | 135,302 | 0.56 | 37.0 | 0 | 79 |  | 16.8 | -2.5 | 37.2 |
| Las Palmas | 118,825 | 1.09 | 54.2 | 0 | 162 |  | 24.7 | 15.8 | 36.2 |  | 401,323 | 1.66 | 109.9 | 0 | 168 |  | 24.2 | 16.0 | 39.0 |
| Pontevedra | 247,938 | 2.28 | 113.2 | 0 | 203 |  | 19.3 | 4.6 | 36.4 |  | 583,130 | 2.41 | 159.6 | 0 | 242 |  | 19.1 | 4.5 | 39.5 |
| Salamanca | 121,594 | 1.12 | 55.5 | 0 | 115 |  | 19.0 | -3.0 | 37.4 |  | 201,733 | 0.84 | 55.2 | 0 | 110 |  | 20.0 | 1.0 | 39.7 |
| Santa Cruz de Tenerife | 118,442 | 1.09 | 54.1 | 0 | 142 |  | 24.8 | 16.4 | 36.3 |  | 347,670 | 1.44 | 95.2 | 0 | 154 |  | 25.0 | 15.4 | 42.9 |
| Cantabria | 140,168 | 1.29 | 64 | 0 | 120 |  | 18.7 | 4.7 | 35.8 |  | 311,137 | 1.29 | 85.2 | 0 | 139 |  | 18.8 | 4.0 | 37.8 |
| Segovia | 45,745 | 0.42 | 20.9 | 0 | 40 |  | 18.1 | -1.8 | 36.5 |  | 89,825 | 0.37 | 24.6 | 0 | 49 |  | 18.1 | -2.6 | 38.1 |
| Sevilla | 361,662 | 3.33 | 165.1 | 0 | 329 |  | 25.6 | 10.2 | 41.8 |  | 801,126 | 3.32 | 219.3 | 0 | 330 |  | 25.8 | 4.0 | 45.9 |
| Soria | 28,234 | 0.26 | 12.9 | 0 | 39 |  | 17.5 | -1.0 | 35.8 |  | 63,735 | 0.26 | 17.4 | 0 | 38 |  | 17.4 | -4.0 | 36.6 |
| Tarragona | 178,903 | 1.65 | 81.7 | 0 | 147 |  | 21.7 | 2.2 | 36.4 |  | 424,379 | 1.76 | 116.2 | 0 | 195 |  | 21.4 | 4.3 | 38.0 |
| Teruel | 40,855 | 0.38 | 18.6 | 0 | 40 |  | 19.7 | -4.8 | 37.5 |  | 90,078 | 0.37 | 24.7 | 0 | 48 |  | 19.8 | -2.4 | 40.2 |
| Toledo | 169,787 | 1.56 | 77.5 | 0 | 149 |  | 22.3 | -0.4 | 41.2 |  | 364,058 | 1.51 | 99.7 | 0 | 161 |  | 22.5 | -0.2 | 43.1 |
| Valencia | 597,125 | 5.49 | 272.5 | 0 | 465 |  | 23.4 | 5.8 | 40.5 |  | 1,324,593 | 5.49 | 362.6 | 0 | 545 |  | 23.0 | 3.9 | 42.8 |
| Valladolid | 169,972 | 1.56 | 77.6 | 0 | 141 |  | 19.0 | -1.4 | 38.3 |  | 327,483 | 1.36 | 89.6 | 0 | 169 |  | 18.8 | -2.0 | 39.4 |
| Vizcaya | 315,967 | 2.91 | 144.2 | 0 | 237 |  | 19.4 | 4.2 | 37.3 |  | 641,597 | 2.66 | 175.6 | 0 | 285 |  | 19.5 | 2.1 | 41.0 |
| Zamora | 63,548 | 0.58 | 29 | 0 | 64 |  | 19.1 | -1.0 | 37.4 |  | 143,829 | 0.6 | 39.4 | 0 | 75 |  | 19.3 | -2.6 | 39.1 |
| Zaragoza | 251,931 | 2.32 | 115 | 0 | 194 |  | 21.4 | -0.2 | 40.5 |  | 541,985 | 2.24 | 148.4 | 0 | 233 |  | 21.5 | -3.0 | 43.1 |
