# Supplementary material for: Temporal changes in the effects of ambient temperatures on hospital admissions in Spain
Source: PLoS One. 2019 Jun 13;14(6):e0218262. doi: 10.1371/journal.pone.0218262 (PMC6564013; doi:10.1371/journal.pone.0218262)
Supplement: S5 Table — MHP: Minimum Hospitalizations Percentile. Models for respiratory diseases were not control for influenza epidemics. *p_value<0.05. (DOCX) [file pone.0218262.s005.docx]

# S5 Table: Percent change (%) and 95% confidence intervals for the relationship between cold and heat and hospitalizations in Spain for sex, age and cause of hospitalization for the period 1997-2013, excluding year 2003.

|  | **Cold** | | **Heat** | **MHP** |
| --- | --- | --- | --- | --- |
| **Cardiovascular diseases** | | |  |  |
| Total | 33 (29,38)* | | -11 (-14,-9)* | 90 |
| Sex |  | |  |  |
| Women | 41 (35,47)* | | -14 (-17,-10)* | 90 |
| Men | 27 (22,32)* | | -10 (-13,-6)* | 82 |
| Age |  | |  |  |
| 16-64 | 21 (15,26)* | | -6 (-10,-1)* | 78 |
| 65-74 | 30 (21,40)* | | -13 (-17,-8)* | 90 |
| 75-84 | 38 (31,45)* | | -16 (-19,-12)* | 90 |
| >=85 | 44 (35,53)* | | -11 (-16,-6)* | 90 |
|  |  | |  |  |
| **Cerebrovascular diseases** | | |  |  |
| Total | 32 (26,39)* | | -2 (-7,4) | 78 |
| Sex |  | |  |  |
| Women | 43 (34,53)* | | 0 (-8,8) | 90 |
| Men | 28 (18,39)* | | -1 (-8,7) | 68 |
| Age |  | |  |  |
| 16-64 | 17 (7,28)* | | 1 (-8,12) | 72 |
| 65-74 | 31 (18,45)* | | -5 (-13,4) | 86 |
| 75-84 | 41 (32,51)* | | -2 (-9,5) | 72 |
| >=85 | 38 (24,54)* | | -5 (-14,4) | 90 |
|  |  | |  |  |
| **Respiratory diseases** | | |  |  |
| Total | 38 (31,45)* | | 8 (4,12)* | 77 |
| Sex |  | |  |  |
| Women | 38 (31,46)* | | 10 (3,17)* | 76 |
| Men | 38 (31,45)* | | 7 (2,11)* | 78 |
| Age |  | |  |  |
| 16-64 | 47 (37,57)* | 2 (-5,10) | | 77 |
| 65-74 | 43 (33,55)* | 8 (1,15)* | | 89 |
| 75-84 | 38 (29,47)* | 11 (5,18)* | | 89 |
| >=85 | 43 (33,54)* | 22 (11,34)* | | 62 |

MHP: Minimum Hospitalizations Percentile

Models for respiratory diseases were not control for influenza epidemics.

*p_value<0.05
