# Supplementary material for: Temporal changes in the effects of ambient temperatures on hospital admissions in Spain
Source: PLoS One. 2019 Jun 13;14(6):e0218262. doi: 10.1371/journal.pone.0218262 (PMC6564013; doi:10.1371/journal.pone.0218262)
Supplement: S6 Table — MHP: Minimum Hospitalizations Percentile. Models for respiratory diseases were not control for influenza epidemics. Some provinces were excluded from the model due to convergence problems. (a) 1 province; (b) 1 province; (c) 1 province; (d) 4 provinces; (e) 1 province; (f) 1 province; (g) 4 provinces; (h) 1 province. *p-value<0.05. (DOCX) [file pone.0218262.s006.docx]

S6 Table: Percent Change (%) and 95% Confidence Intervals for the relationship between cold and heat and mortality in Spain for 1997-2004 (Period 1) and 2005-2013 (Period 2).

|  | **PERIOD 1 (1997-2004)** | |  |  | **PERIOD 2 (2005-2013)** | | |
| --- | --- | --- | --- | --- | --- | --- | --- |
|  | **COLD** | **HEAT** | **MHP** |  | **COLD** | **HEAT** | **MHP** |
| **Cardiovascular diseases** | |  |  |  |  |  |  |
| **Total** | **28 (21,35)*** | **-5 (-8,-2)*** | **85** |  | **44 (38,50)*** | **- 11 (-14,-9)*** | **90** |
| Sex |  |  |  |  |  |  |  |
| Women | 38 (28,48)* | -8 (-12,-4)* | 90 |  | 49 (42,57)* | -13 (-16,-9)* | 90 |
| Men | 23 (17,31)* | 0 (-6,7) | 67 |  | 38 (31,45)* | -11 (-14,-7)* | 90 |
| Age |  |  |  |  |  |  |  |
| 16-64 | 19 (11,27)* | 2 (-7,10) | 72 |  | 27 (19,36)* | -7 (-13,-2)* | 87 |
| 65-74^(a)^ | 30 (19,42)* | -8 (-13,-3)* | 90 |  | 41 (30,54)* | -13 (-19,-8)* | 90 |
| 75-84^(b)^ | 31 (20,43)* | -7 (-13,0) | 77 |  | 50 (41,59)* | -14 (-19,-10)* | 90 |
| >=85^(c)^ | 30 (19,43)* | -6 (-17,6) | 63 |  | 58 (46,72)* | -9 (-15,3) | 90 |
| **Cerebrovascular diseases** | |  |  |  |  |  |  |
| **Total** | **32 (23,42)*** | **2 (-5,9)** | **75** |  | **38 (29,48)*** | **-3 (-9,3)** | **90** |
| Sex |  |  |  |  |  |  |  |
| Women | 42 (25,62)* | 9 (-1,19) | 90 |  | 47 (34,60)* | -6 (-13,3) | 90 |
| Men | 25 (15,35)* | -3 (-1,19) | 66 |  | 35 (22,49)* | 0 (-9,9) | 71 |
| Age |  |  |  |  |  |  |  |
| 16-64 | 22 (7,40)* | -2 (-15,11) | 72 |  | 12 (0,25)* | 1 (-11,15) | 68 |
| 65-74^(d)^ | 23 (8,39)* | -3 (-14,10) | 81 |  | 39 (20,62)* | -8 (-18,2) | 90 |
| 75-84 | 41 (28,55)* | 6 (-5,18) | 69 |  | 47 (33,62)* | -5 (-13,4) | 80 |
| >=85^(e)^ | 31 (8,58)* | 3 (-3,22) | 90 |  | 45 (28,65)* | -9 (-19,3) | 86 |
| **Respiratory diseases** | |  |  |  |  |  |  |
| **Total^(f)^** | **48 (39,57)*** | **12 (4,19)*** | **48** |  | **48 (39,56)*** | **5 (1,10)*** | **82** |
| Sex |  |  |  |  |  |  |  |
| Women | 48 (35,63)* | 13 (3,25)* | 43 |  | 50 (39,62)* | 9 (2,17)* | 76 |
| Men | 48 (36,61)* | 10 (4,17)* | 89 |  | 46  (39,55)* | 3 (-2,8) | 87 |
| Age |  |  |  |  |  |  |  |
| 16-64 | 48 (30,68)* | 6 (-3,16) | 90 |  | 56 (44,68)* | -3 (-10,6) | 84 |
| 65-74^(g)^ | 60 (42,79)* | 9 (-1,19) | 90 |  | 54 (40,70)* | 3 (-4,12) | 84 |
| 75-84^(h)^ | 59 (42,78)* | 18 (9,28)* | 90 |  | 50 (38,62)* | 8 (1,15)* | 90 |
| >=85 | 64 (47,84)* | 40 (21,61)* | 43 |  | 57 (44,71)* | 20 (10,31)* | 77 |

MHP: Minimum Hospitalizations Percentile

Models for respiratory diseases were not control for influenza epidemics. Some provinces were excluded from the model due to convergence problems. (a) 1 province; (b) 1 province; (c) 1 province; (d) 4 provinces; (e) 1 province; (f) 1 province; (g) 4 provinces; (h) 1 province

*p-value<0.05
