# Supplementary material for: Temporal changes in the effects of ambient temperatures on hospital admissions in Spain
Source: PLoS One. 2019 Jun 13;14(6):e0218262. doi: 10.1371/journal.pone.0218262 (PMC6564013; doi:10.1371/journal.pone.0218262)
Supplement: S7 Table — Activation HHPP: thresholds established in each province to activate the Spanish Heat Health Prevention Plan. (DOCX) [file pone.0218262.s007.docx]

# S7 Table: Number of days in which the Spanish Heat Health Prevention Plan was activated and number of days considered heat wave, according to the different definitions, by periods (1997-2002 and 2004-2013) and Spanish provinces

|  | **PERIOD 1 (1997-2002)** | | | | | | | | | | | | | |  | **PERIOD 2 (2004-2013)** | | | | | | | | | | | | | |
| --- | --- | --- | --- | --- | --- | --- | --- | --- | --- | --- | --- | --- | --- | --- | --- | --- | --- | --- | --- | --- | --- | --- | --- | --- | --- | --- | --- | --- | --- |
| **Province** | **Activation HHPP** | **Mean HHPP / year** | **≥2 d + 90th p.** | **≥2 d + 92.5th p.** | **≥2 d + 95th p.** | **≥2 d + 97.5th p.** | **≥3 d + 90th p.** | **≥3 d + 92.5th p.** | **≥3 d + 95th p.** | **≥3 d + 97.5th p.** | **≥4 d + 90th p.** | **≥4 d + 92.5th p.** | **≥4 d + 95th p.** | **≥4 d + 97.5th p.** |  | **Activation HHPP** | **Mean HHPP / year** | **≥2 d + 90th p.** | **≥2 d + 92.5th p.** | **≥2 d + 95th p.** | **≥2 d + 97.5th p.** | **≥3 d + 90th p.** | **≥3 d + 92.5th p.** | **≥3 d + 95th p.** | **≥3 d + 97.5th p.** | **≥4 d + 90th p.** | **≥4 d + 92.5th p.** | **≥4 d + 95th p.** | **≥4 d + 97.5th p.** |
| Alava | 0 | 0 | 19 | 12 | 7 | 4 | 6 | 4 | 3 | 3 | 2 | 2 | 2 | 2 |  | 5 | 0.5 | 45 | 37 | 21 | 13 | 15 | 12 | 6 | 3 | 4 | 3 | 2 | 1 |
| Albacete | 39 | 6.5 | 32 | 24 | 11 | 2 | 14 | 10 | 4 | 0 | 5 | 5 | 3 | 0 |  | 121 | 12.1 | 63 | 44 | 27 | 10 | 30 | 20 | 10 | 1 | 14 | 8 | 2 | 0 |
| Alicante | 10 | 1.7 | 20 | 12 | 4 | 3 | 8 | 5 | 2 | 2 | 4 | 2 | 1 | 1 |  | 15 | 1.5 | 29 | 20 | 9 | 3 | 7 | 5 | 0 | 0 | 1 | 1 | 0 | 0 |
| Almeria | 70 | 11.7 | 32 | 22 | 12 | 6 | 16 | 8 | 2 | 1 | 10 | 3 | 0 | 0 |  | 158 | 15.8 | 33 | 19 | 10 | 2 | 13 | 3 | 0 | 0 | 5 | 0 | 0 | 0 |
| Avila | 0 | 0.0 | 35 | 21 | 11 | 0 | 17 | 8 | 3 | 0 | 7 | 3 | 0 | 0 |  | 0 | 0 | 66 | 44 | 28 | 13 | 29 | 21 | 8 | 4 | 11 | 9 | 2 | 0 |
| Badajoz | 25 | 4.2 | 28 | 15 | 6 | 2 | 9 | 2 | 0 | 0 | 3 | 0 | 0 | 0 |  | 86 | 8.6 | 80 | 52 | 25 | 10 | 42 | 22 | 8 | 3 | 23 | 9 | 0 | 0 |
| Illes Balears | 10 | 1.7 | 27 | 16 | 11 | 4 | 12 | 4 | 2 | 1 | 4 | 0 | 0 | 0 |  | 31 | 3.1 | 48 | 38 | 27 | 6 | 22 | 18 | 9 | 1 | 10 | 7 | 3 | 0 |
| Barcelona | 21 | 3.5 | 9 | 6 | 3 | 1 | 2 | 2 | 1 | 0 | 0 | 0 | 0 | 0 |  | 223 | 22.3 | 81 | 57 | 37 | 13 | 46 | 33 | 19 | 4 | 29 | 21 | 10 | 2 |
| Burgos | 0 | 0.0 | 29 | 22 | 13 | 6 | 9 | 8 | 6 | 4 | 3 | 2 | 2 | 2 |  | 5 | 0.5 | 51 | 35 | 17 | 6 | 22 | 13 | 3 | 0 | 11 | 6 | 0 | 0 |
| Caceres | 20 | 3.3 | 30 | 22 | 11 | 2 | 17 | 9 | 4 | 0 | 8 | 3 | 2 | 0 |  | 96 | 9.6 | 69 | 49 | 31 | 19 | 30 | 20 | 13 | 6 | 14 | 7 | 4 | 1 |
| Cadiz | 11 | 1.8 | 15 | 6 | 3 | 2 | 4 | 0 | 0 | 0 | 2 | 0 | 0 | 0 |  | 118 | 11.8 | 69 | 51 | 31 | 13 | 36 | 28 | 16 | 5 | 16 | 13 | 5 | 2 |
| Castellon | 5 | 0.8 | 14 | 9 | 6 | 3 | 5 | 3 | 2 | 0 | 1 | 1 | 1 | 0 |  | 56 | 5.6 | 67 | 42 | 15 | 3 | 36 | 16 | 2 | 0 | 22 | 5 | 0 | 0 |
| Ciudad Real | 131 | 21.8 | 31 | 21 | 11 | 2 | 12 | 7 | 2 | 0 | 2 | 2 | 0 | 0 |  | 240 | 24 | 60 | 39 | 22 | 11 | 28 | 15 | 8 | 3 | 12 | 4 | 1 | 0 |
| Cordoba | 15 | 2.5 | 24 | 9 | 4 | 2 | 12 | 4 | 2 | 0 | 6 | 2 | 0 | 0 |  | 68 | 6.8 | 71 | 48 | 34 | 17 | 36 | 20 | 13 | 4 | 14 | 7 | 3 | 0 |
| A Coruna | 5 | 0.8 | 16 | 10 | 7 | 4 | 6 | 3 | 3 | 1 | 2 | 1 | 1 | 0 |  | 5 | 0.5 | 52 | 45 | 29 | 8 | 25 | 22 | 11 | 2 | 11 | 9 | 3 | 0 |
| Cuenca | 36 | 6.0 | 23 | 15 | 3 | 0 | 8 | 3 | 0 | 0 | 2 | 0 | 0 | 0 |  | 81 | 8.1 | 79 | 59 | 35 | 20 | 44 | 28 | 12 | 7 | 23 | 10 | 2 | 1 |
| Girona | 10 | 1.7 | 12 | 11 | 4 | 1 | 5 | 4 | 1 | 0 | 2 | 1 | 0 | 0 |  | 73 | 7.3 | 82 | 60 | 40 | 18 | 54 | 36 | 21 | 10 | 37 | 24 | 11 | 6 |
| Granada | 0 | 0.0 | 26 | 13 | 8 | 5 | 9 | 5 | 2 | 1 | 1 | 0 | 0 | 0 |  | 5 | 0.5 | 78 | 58 | 38 | 12 | 48 | 32 | 18 | 2 | 26 | 17 | 7 | 0 |
| Guadalajara | 0 | 0.0 | 32 | 21 | 12 | 1 | 13 | 8 | 5 | 0 | 3 | 2 | 1 | 0 |  | 17 | 1.7 | 54 | 42 | 28 | 15 | 31 | 21 | 12 | 7 | 18 | 10 | 4 | 2 |
| Guipuzcoa | 0 | 0.0 | 22 | 11 | 7 | 4 | 7 | 3 | 2 | 2 | 1 | 1 | 1 | 1 |  | 0 | 0 | 31 | 17 | 12 | 3 | 10 | 5 | 3 | 0 | 2 | 0 | 0 | 0 |
| Huelva | 25 | 4.2 | 15 | 10 | 8 | 1 | 6 | 4 | 4 | 0 | 3 | 2 | 2 | 0 |  | 86 | 8.6 | 65 | 45 | 30 | 18 | 34 | 22 | 16 | 8 | 16 | 10 | 7 | 4 |
| Huesca | 69 | 11.5 | 26 | 16 | 13 | 3 | 14 | 8 | 7 | 0 | 9 | 4 | 4 | 0 |  | 197 | 19.7 | 67 | 50 | 26 | 14 | 35 | 26 | 13 | 8 | 15 | 12 | 5 | 3 |
| Jaen | 5 | 0.8 | 21 | 11 | 3 | 1 | 4 | 2 | 0 | 0 | 0 | 0 | 0 | 0 |  | 37 | 3.7 | 70 | 54 | 37 | 17 | 42 | 28 | 15 | 7 | 22 | 11 | 6 | 2 |
| Leon | 5 | 0.8 | 38 | 27 | 14 | 6 | 14 | 11 | 5 | 4 | 7 | 5 | 2 | 2 |  | 5 | 0.5 | 58 | 41 | 20 | 10 | 27 | 18 | 6 | 2 | 11 | 8 | 2 | 0 |
| Lleida | 5 | 0.8 | 32 | 28 | 12 | 6 | 20 | 15 | 4 | 2 | 12 | 8 | 1 | 1 |  | 40 | 4 | 63 | 55 | 27 | 14 | 35 | 28 | 12 | 6 | 19 | 13 | 5 | 2 |
| La Rioja | 5 | 0.8 | 26 | 19 | 13 | 5 | 10 | 8 | 5 | 3 | 5 | 4 | 3 | 1 |  | 6 | 0.6 | 54 | 42 | 21 | 12 | 21 | 16 | 8 | 3 | 8 | 6 | 2 | 0 |
| Lugo | 0 | 0.0 | 18 | 16 | 9 | 5 | 9 | 7 | 4 | 3 | 2 | 2 | 1 | 1 |  | 0 | 0 | 53 | 36 | 25 | 7 | 22 | 12 | 7 | 2 | 6 | 2 | 2 | 0 |
| Madrid | 5 | 0.8 | 19 | 11 | 6 | 2 | 9 | 4 | 2 | 0 | 2 | 1 | 0 | 0 |  | 65 | 6.5 | 75 | 55 | 32 | 13 | 43 | 28 | 13 | 3 | 21 | 12 | 3 | 0 |
| Malaga | 88 | 14.7 | 17 | 8 | 5 | 2 | 4 | 2 | 1 | 0 | 0 | 0 | 0 | 0 |  | 169 | 16.9 | 37 | 26 | 17 | 4 | 8 | 5 | 2 | 1 | 2 | 1 | 1 | 0 |
| Murcia | 20 | 3.3 | 9 | 6 | 4 | 0 | 4 | 4 | 3 | 0 | 2 | 2 | 2 | 0 |  | 145 | 14.5 | 39 | 27 | 19 | 8 | 12 | 5 | 3 | 2 | 2 | 0 | 0 | 0 |
| Navarra | 0 | 0.0 | 23 | 20 | 8 | 4 | 8 | 7 | 3 | 2 | 3 | 3 | 1 | 1 |  | 10 | 1 | 55 | 41 | 22 | 13 | 19 | 12 | 6 | 5 | 7 | 2 | 2 | 2 |
| Ourense | 0 | 0.0 | 24 | 14 | 7 | 6 | 11 | 7 | 4 | 4 | 6 | 5 | 3 | 3 |  | 0 | 0 | 62 | 45 | 28 | 12 | 31 | 20 | 11 | 3 | 17 | 12 | 7 | 1 |
| Asturias | 0 | 0.0 | 21 | 16 | 9 | 5 | 5 | 5 | 3 | 2 | 0 | 0 | 0 | 0 |  | 0 | 0 | 30 | 23 | 9 | 4 | 17 | 11 | 2 | 0 | 8 | 4 | 0 | 0 |
| Palencia | 0 | 0.0 | 29 | 22 | 13 | 6 | 9 | 8 | 6 | 4 | 3 | 2 | 2 | 2 |  | 5 | 0.5 | 51 | 35 | 17 | 6 | 22 | 13 | 3 | 0 | 11 | 6 | 0 | 0 |
| Las Palmas | 10 | 1.7 | 19 | 12 | 6 | 0 | 6 | 4 | 2 | 0 | 1 | 1 | 0 | 0 |  | 74 | 7.4 | 83 | 62 | 38 | 21 | 52 | 35 | 20 | 11 | 30 | 19 | 11 | 5 |
| Pontevedra | 0 | 0.0 | 27 | 18 | 10 | 1 | 15 | 6 | 3 | 0 | 10 | 1 | 0 | 0 |  | 11 | 1.1 | 68 | 45 | 30 | 15 | 38 | 23 | 16 | 6 | 22 | 14 | 9 | 2 |
| Salamanca | 0 | 0.0 | 19 | 11 | 9 | 2 | 8 | 5 | 4 | 1 | 4 | 2 | 2 | 0 |  | 15 | 1.5 | 72 | 47 | 25 | 9 | 31 | 15 | 9 | 0 | 13 | 4 | 2 | 0 |
| Santa Cruz de Tenerife | 10 | 1.7 | 23 | 10 | 3 | 0 | 13 | 4 | 1 | 0 | 7 | 2 | 0 | 0 |  | 110 | 11 | 89 | 64 | 47 | 23 | 57 | 36 | 26 | 13 | 37 | 20 | 14 | 7 |
| Cantabria | 0 | 0.0 | 16 | 11 | 4 | 3 | 3 | 2 | 1 | 1 | 1 | 1 | 0 | 0 |  | 0 | 0 | 50 | 34 | 17 | 5 | 25 | 14 | 5 | 2 | 14 | 9 | 4 | 1 |
| Segovia | 24 | 4.0 | 29 | 20 | 9 | 5 | 14 | 9 | 4 | 2 | 5 | 3 | 2 | 1 |  | 94 | 9.4 | 60 | 46 | 19 | 10 | 26 | 19 | 6 | 3 | 10 | 7 | 0 | 0 |
| Sevilla | 15 | 2.5 | 19 | 9 | 5 | 0 | 9 | 1 | 0 | 0 | 5 | 0 | 0 | 0 |  | 119 | 11.9 | 87 | 55 | 37 | 17 | 49 | 30 | 16 | 5 | 24 | 13 | 4 | 2 |
| Soria | 0 | 0.0 | 38 | 25 | 16 | 6 | 18 | 12 | 6 | 3 | 8 | 6 | 4 | 1 |  | 0 | 0 | 59 | 44 | 18 | 7 | 25 | 21 | 5 | 2 | 9 | 6 | 1 | 0 |
| Tarragona | 10 | 1.7 | 20 | 11 | 6 | 2 | 9 | 6 | 2 | 0 | 4 | 2 | 0 | 0 |  | 25 | 2.5 | 65 | 41 | 17 | 6 | 42 | 24 | 8 | 2 | 28 | 14 | 4 | 0 |
| Teruel | 0 | 0.0 | 27 | 17 | 7 | 1 | 14 | 7 | 3 | 0 | 7 | 3 | 2 | 0 |  | 0 | 0 | 60 | 50 | 29 | 11 | 26 | 22 | 11 | 2 | 8 | 7 | 1 | 0 |
| Toledo | 71 | 11.8 | 33 | 24 | 13 | 1 | 14 | 8 | 4 | 0 | 2 | 2 | 1 | 0 |  | 120 | 12 | 61 | 41 | 24 | 8 | 30 | 16 | 6 | 2 | 14 | 6 | 0 | 0 |
| Valencia | 20 | 3.3 | 14 | 8 | 5 | 1 | 4 | 2 | 1 | 0 | 1 | 0 | 0 | 0 |  | 35 | 3.5 | 52 | 42 | 15 | 3 | 30 | 22 | 2 | 0 | 22 | 17 | 1 | 0 |
| Valladolid | 5 | 0.8 | 35 | 21 | 12 | 6 | 14 | 10 | 4 | 3 | 8 | 5 | 2 | 2 |  | 5 | 0.5 | 64 | 36 | 18 | 8 | 31 | 12 | 6 | 0 | 17 | 5 | 1 | 0 |
| Vizcaya | 0 | 0.0 | 15 | 10 | 6 | 1 | 6 | 5 | 2 | 0 | 2 | 2 | 1 | 0 |  | 5 | 0.5 | 39 | 22 | 12 | 4 | 15 | 5 | 3 | 0 | 6 | 2 | 0 | 0 |
| Zamora | 0 | 0.0 | 22 | 12 | 8 | 4 | 8 | 3 | 2 | 1 | 3 | 1 | 0 | 0 |  | 5 | 0.5 | 70 | 50 | 27 | 12 | 34 | 22 | 12 | 3 | 17 | 7 | 4 | 1 |
| Zaragoza | 11 | 1.8 | 19 | 14 | 6 | 3 | 8 | 5 | 2 | 1 | 2 | 2 | 1 | 0 |  | 90 | 9 | 65 | 44 | 26 | 11 | 31 | 18 | 10 | 2 | 17 | 8 | 4 | 0 |
| **TOTAL** | **811** | **135** | **1,169** | **755** | **403** | **142** | **481** | **281** | **138** | **51** | **192** | **101** | **50** | **21** |  | **2,876** | **288** | **3,031** | **2,154** | **1,245** | **537** | **1,514** | **968** | **470** | **165** | **756** | **427** | **161** | **47** |

Activation HHPP: thresholds established in each province to activate the Spanish Heat Health Prevention Plan
