# Supplementary material for: Temporal changes in the effects of ambient temperatures on hospital admissions in Spain
Source: PLoS One. 2019 Jun 13;14(6):e0218262. doi: 10.1371/journal.pone.0218262 (PMC6564013; doi:10.1371/journal.pone.0218262)
Supplement: S8 Table — Some provinces were excluded from the model due to a high variability and unstable results: (a) 1 province; (b) 1 province, (c) 1 province, (d) 1 province. *p-value<0.05. (DOCX) [file pone.0218262.s008.docx]

S8 Table: Percent Change (%) and 95% Confidence Intervals for different definitions of heat waves in the two study periods (1997-2002 and 2004-2013).

|  | **Nº DAYS** | |  | **CARDIOVASCULAR DISEASES** | |  | **CEREBROVASCULAR DISEASES** | |  | **RESPIRATORY DISEASES** | |
| --- | --- | --- | --- | --- | --- | --- | --- | --- | --- | --- | --- |
|  | **PERIOD 1** | **PERIOD 2** |  | **PERIOD 1** | **PERIOD 2** |  | **PERIOD 1** | **PERIOD 2** |  | **PERIOD 1** | **PERIOD 2** |
| ≥2 days + 90th percentile | 1,169 | 3,031 |  | -3 (-5,-1)* | -2 (-3,-1)* |  | -1 (-4,4) | 0 (-2,2) |  | 5 (3,8)* | 5 (3,6)* |
| ≥2 days + 92.5th percentile | 755 | 2,154 |  | -2 (-5,1) | -3 (-4,-1)* |  | 1 (-4,6) | 0 (-2,2) |  | 7 (3,11)* | 5 (3,7)* |
| ≥2 days + 95th percentile | 403 | 1,245 |  | -1 (-5,3) | -2 (-4,-1)* |  | 5 (-2,13) | 0 (-3,3) |  | 10 (4,16)* | 4 (2,7)* |
| ≥2 days + 97.5th percentile | 142 | 537 |  | -4 (-10,2) | -2 (-5,1) |  | 9 (-3,22) ^(b)^ | -1 (-6,4) |  | 12 (3,21)* | 4 (1,7)* |
| ≥3 days + 90th percentile | 481 | 1,514 |  | -3 (-6,1) | -2 (-3,0) |  | 2 (-4,9) | 0 (-3,2) |  | 12 (8,17)* | 7 (5,9)* |
| ≥3 days + 92.5th percentile | 281 | 968 |  | -3 (-8,2) | -2 (-4,0) |  | 3 (-6,13) | 1 (-2,4) |  | 15 (7,22)* | 8 (4,11)* |
| ≥3 days + 95th percentile | 138 | 470 |  | 2 (-4,9) | -1 (-4,1) |  | 3 (-10,17) | 4 (-1,9) |  | 19 (9,30)* | 9 (4,14)* |
| ≥3 days + 97.5th percentile | 51 | 165 |  | 5 (-10,23) | 3 (-8,14) |  | 16 (-9,47) | 15 (-8,44) |  | 22 (5,43)* | 8 (-4,21) |
| ≥4 days + 90th percentile | 192 | 756 |  | -4 (-9,2) | -3 (-5,-1)* |  | 4 (-5,14) | -1 (-5,4) |  | 17 (10,24)* | 9 (5,12)* |
| ≥4 days + 92.5th percentile | 101 | 427 |  | -1 (-9,8) ^(a)^ | -3 (-7,0) |  | 10 (-3,26) | 2 (-4,9) |  | 20 (10,31)* | 11 (5,17)* ^(c)^ |
| ≥4 days + 95th percentile | 50 | 161 |  | 4 (-10,21) | -4 (-13,5) |  | 3 (-21,33) | 0 (-16,19) |  | 38 (19,59)* | 9 (-2,21) ^(d)^ |
| ≥4 days + 97.5th percentile | 21 | 47 |  | -12 (-40,27) | -11 (-33,20) |  | 7 (-37,82) | -9 (-68,163) |  | 29 (-6,77)* | -1 (-26,34) |

Some provinces were excluded from the model due to a high variability and unstable results: (a) 1 province; (b) 1 province, (c) 1 province, (d) 1 province.

*p-value<0.05
