# Supplementary material for: Temporal changes in the effects of ambient temperatures on hospital admissions in Spain
Source: PLoS One. 2019 Jun 13;14(6):e0218262. doi: 10.1371/journal.pone.0218262 (PMC6564013; doi:10.1371/journal.pone.0218262)
Supplement: S9 Table — Df: degrees of freedom. *p-value<0.05. (DOCX) [file pone.0218262.s009.docx]

# S9 Table: Sensitivity analyses

**Percent Change (%) and 95% Confident Intervals for the relationship between cold and heat and mortality in Spain for the Period 1 (1997-2002) and Period 2 (2004-2013)**

| **Modelling choices** | **CARDIOVASCULAR DISEASES** | |  | **CEREBROVASCULAR DISEASES** | |  | **RESPIRATORY DISEASES** | |
| --- | --- | --- | --- | --- | --- | --- | --- | --- |
|  | **COLD** | **HEAT** |  | **COLD** | **HEAT** |  | **COLD** | **HEAT** |
| Main model | 34 (29,38)* | -8 (-10,-6)* |  | 32 (26,38)* | 2 (-3,7) |  | 38 (31,45)* | 14 (9,19)* |
| Lag period: 10 | 15 (12,18)* | -4 (-6,-3)* |  | 29 (24,34)* | 1 (-1,4) |  | 10 (7,13)* | 22 (18,26)* |
| Lag period: 30 | 41 (35,47)* | -5 (-9,-2)* |  | 48 (37,59)* | 5 (-3,13) |  | 79 (67,92)* | 15 (9,22)* |
| Df/year for seasonal control: 6 | 43 (38,48)* | 16 (12,20)* |  | 48 (40,57)* | 24 (19,29)* |  | 67 (56,79)* | 41 (34,47)* |
| Df/year for seasonal control: 10 | 42 (37,47)* | -5 (-8,-3)* |  | 36 (29,44)* | 6 (1,11)* |  | 73 (58,90)* | 6 (1,13)* |

Df: degrees of freedom

*p-value<0.05
