# Supplementary figures and images for: Temporal changes in the effects of ambient temperatures on hospital admissions in Spain
Source: PLoS One. 2019 Jun 13;14(6):e0218262. doi: 10.1371/journal.pone.0218262 (PMC6564013; doi:10.1371/journal.pone.0218262)

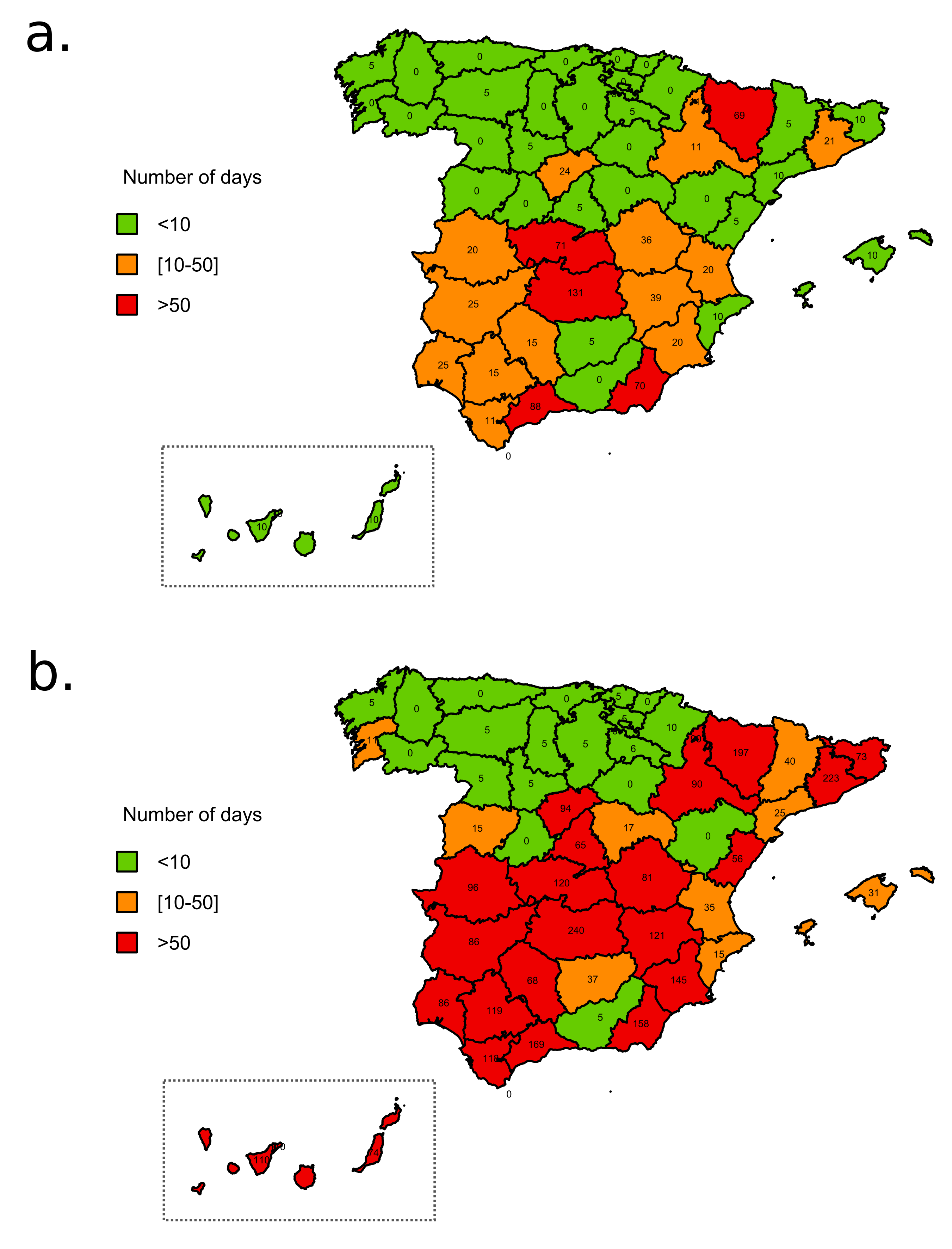

Supplement: S2 Fig — Number of days for the activation of the Spanish Heat Health Prevention Plan by provinces in the two study periods: a) period 1 (1997–2002) and b) period 2 (2004–2013). (TIF) [file pone.0218262.s011.tif]
